# The Planar Polarity Component VANGL2 Is a Key Regulator of Mechanosignaling

**DOI:** 10.3389/fcell.2020.577201

**Published:** 2020-10-29

**Authors:** Sek-Shir Cheong, Khondoker M. Akram, Carlos Matellan, Sally Yunsun Kim, David C. A. Gaboriau, Matthew Hind, Armando E. del Río Hernández, Mark Griffiths, Charlotte H. Dean

**Affiliations:** ^1^National Heart and Lung Institute, Imperial College London, London, United Kingdom; ^2^Cellular and Molecular Biomechanics Laboratory, Department of Bioengineering, Imperial College London, London, United Kingdom; ^3^Facility for Imaging by Light Microscopy, National Heart and Lung Institute, Faculty of Medicine, Imperial College London, London, United Kingdom; ^4^National Institute for Health Research, Respiratory Biomedical Research Unit, Royal Brompton & Harefield NHS Foundation Trust, London, United Kingdom; ^5^Peri-Operative Medicine Department, St Bartholomew’s Hospital, London, United Kingdom; ^6^MRC Harwell Institute, Harwell Campus, Oxfordshire, United Kingdom

**Keywords:** VANGL2, planar cell polarity, mechanosignaling, cell migration, focal adhesion, traction force, RhoA, YAP signaling

## Abstract

VANGL2 is a component of the planar cell polarity (PCP) pathway, which regulates tissue polarity and patterning. The *Vangl2*^*Lp*^ mutation causes lung branching defects due to dysfunctional actomyosin-driven morphogenesis. Since the actomyosin network regulates cell mechanics, we speculated that mechanosignaling could be impaired when VANGL2 is disrupted. Here, we used live-imaging of precision-cut lung slices (PCLS) from *Vangl2*^*Lp/+*^ mice to determine that alveologenesis is attenuated as a result of impaired epithelial cell migration. *Vangl2*^*Lp/+*^ tracheal epithelial cells (TECs) and alveolar epithelial cells (AECs) exhibited highly disrupted actomyosin networks and focal adhesions (FAs). Functional assessment of cellular forces confirmed impaired traction force generation in *Vangl2*^*Lp/+*^ TECs. YAP signaling in *Vangl2*^*Lp*^ airway epithelium was reduced, consistent with a role for VANGL2 in mechanotransduction. Furthermore, activation of RhoA signaling restored actomyosin organization in *Vangl2*^*Lp/+*^, confirming RhoA as an effector of VANGL2. This study identifies a pivotal role for VANGL2 in mechanosignaling, which underlies the key role of the PCP pathway in tissue morphogenesis.

## Introduction

The planar cell polarity (PCP) pathway is a non-canonical Wnt pathway, best known for controlling the collective polarization of cells across the plane of a cell sheet, a phenomenon referred to as PCP. In *Drosophila*, the mutually exclusive location of Vangl/Pk and Fz/Dsh/Diego complexes at the proximal and distal side within a cell gives rise to PCP (Goodrich and Strutt, 2011). More broadly, across different organisms, this core set of protein components govern diverse developmental processes through regulation of directed cell migration (DCM) (Henderson et al., 2018). In DCM, groups of cells within the tissue plane adopt multicellular polarization and move in a cohesive manner with the coordination of RhoA-mediated actomyosin contractile activity at the rear and Rac1/Cdc42-induced pushing force at the front, while maintaining cell–cell contacts (Friedl and Gilmour, 2009; Mayor and Carmona-Fontaine, 2010). Our previous studies have shown that the PCP pathway is required for embryonic lung and kidney development (Yates et al., 2010a,b, 2013). More recently, it has been shown that the PCP pathway plays a central role in alveologenesis and adult lung homeostasis (Poobalasingam et al., 2017; Zhang et al., 2020) and is important for normal repair of the lung epithelium following injury (Poobalasingam et al., 2017). Both embryonic and adult PCP mouse mutants display abnormal actomyosin distribution (Yates et al., 2010b; Poobalasingam et al., 2017). Although PCP is known to regulate actin cytoskeleton remodeling through its downstream effector RhoA (Munoz-Soriano et al., 2012; Davey and Moens, 2017; Henderson et al., 2018), the function of the PCP pathway in mechanosignaling, the process by which mechanical cues can rapidly trigger signaling mechanisms that lead to cytoskeletal re-organization and modulation of cell shape or movement, has not been determined.

The actin cytoskeleton consists of polymerized actin filaments (F-actin), which serve as a scaffold for non-muscle myosin II to form the actomyosin contractile system. Mechanical stresses can activate RhoA, which promotes the formation of stress fibers and stimulates the phosphorylation of myosin light chain 2 (MLC2) through Rho-associated kinase (ROCK) (Sun et al., 2016). The intracellular actin cytoskeleton is linked to focal adhesions (FAs) via proteins such as paxillin, talin, and vinculin. These FA complexes mediate bidirectional signaling between cell and extracellular matrix (ECM) via the mechanosensitive transmembrane integrin proteins and the intracellular actin filaments (Mitra et al., 2005; Hopkins et al., 2019). During migration, increased mechanosignaling leads to integrin clustering at FAs, which triggers the autophosphorylation of FA kinase (FAK). This in turn stimulates the recruitment of FA proteins such as paxillin and vinculin to further reinforce the FA maturation (Hamadi et al., 2005; Mitra et al., 2005).

Given the role of the PCP pathway in DCM and our previous observations of disrupted actomyosin network in the lungs (Yates et al., 2010b; Poobalasingam et al., 2017), we hypothesized that VANGL2 could play a key role in mechanosignaling. Interestingly, a previous study described a link between the PCP/WNT5A-Frizzled-DVL pathway with YAP (Park et al., 2015), a mechanotransducer that converts mechanical stimuli into biochemical signals by regulating gene expression in response to mechanical cues (Jaalouk and Lammerding, 2009). Moreover, YAP deficiency caused lung branching defects (Lin et al., 2017), similar to that of *Vangl2*^*Lp*^.

The *Looptail* mutant mouse (*Vangl2*^*Lp*^) carries a missense mutation, S464N, in the *Vangl2* gene. *Vangl2*^*Lp*^ is a dominant negative mutation that not only impairs the transport of mutant VANGL2 from endoplasmic reticulum to the Golgi but also blocks wildtype (WT) VANGL2 protein being trafficked to membrane, thereby resulting in loss of function. The dominant nature of the *Vangl2*^*Lp*^ mutation has been well established in mouse genetic studies and *in vitro* cell systems (Kibar et al., 2001; Murdoch, 2001; Gao et al., 2011; Belotti et al., 2012; Yin et al., 2012; Seo et al., 2017). Although homozygotes die around birth, heterozygotes are viable as adults but display a number of phenotypes including looped tails which led to their name and lung defects (Yates et al., 2010b; Poobalasingam et al., 2017). In addition to disruption of VANGL2, the Looptail mutation also affects additional PCP components such as Prickle2 (Pryor et al., 2014), Frizzled3 (Montcouquiol et al., 2006), and its homolog Vangl1 (Yin et al., 2012) making this a powerful tool for studies of the Wnt/PCP pathway. Thus, using *Vangl2*^*Lp*^, we assessed whether VANGL2 dysfunction affects normal cellular mechanics and mechanosignaling. Real-time imaging of *Vangl2*^*Lp/+*^ precision-cut lung slices (PCLSi) and wound-healing assays with primary lung epithelial cells reveal that VANGL2 is required for normal alveologenesis and wound repair via its role in DCM. We provide mechanistic evidence that VANGL2 disruption affects FA complexes, stress fiber formation, and MLC2 activation, leading to defective intracellular contractility via RhoA signaling. These abnormalities result in impaired traction force generation and deficiency of the mechanotransducer YAP. This study demonstrates that VANGL2 has an important role in mechanosignaling, which underlies the key functions of PCP pathway in regulating tissue morphogenesis and repair.

## Results

### Live Imaging of Alveologenesis in *Vangl2*^*Lp/+*^ PCLS Reveals Impaired Cell Migration

Using the PCLSi technique recently established by our team (Akram et al., 2019a), we investigated whether the alveologenesis defects we previously reported in *Vangl2*^*Lp/+*^ mice were due to impaired cell migration (Poobalasingam et al., 2017).

Precision-cut lung slices (300 μm thick) from WT and *Vangl2*^*Lp/+*^ P3 littermate lungs were labeled with FITC-conjugated epithelial cell-specific marker EpCAM and time-lapse live imaging of *ex vivo* alveologenesis was conducted for 13 h (Supplementary Figure S1A). EpCAM specifically labeled epithelial cells but not macrophages within PCLS, as confirmed by dual staining of EpCAM and CD11c (macrophage marker) (Supplementary Figure S1B). Time-lapse image sequences were subsequently analyzed to track and quantify epithelial cell motility.

**FIGURE 1:**
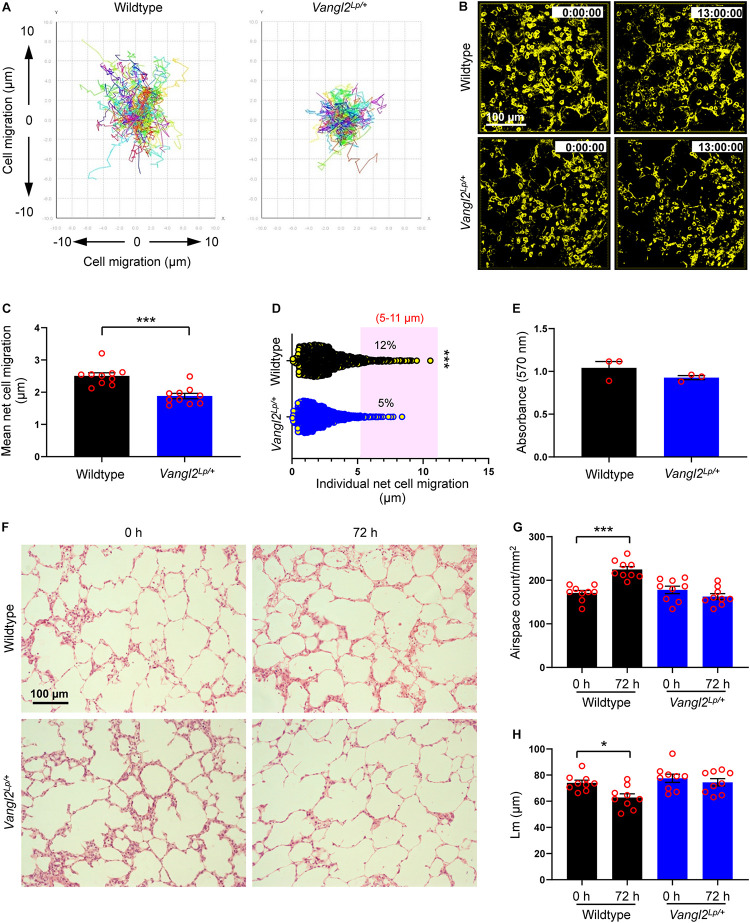
Alveolar epithelial cell migration is disrupted in *Vangl2*^*Lp/+*^ mice during *ex vivo* alveologenesis. **(A)** Traces of individual cell tracking over 13 h in a single field from wildtype and *Vangl2*^*Lp/+*^ P3 mouse PCLS. **(B)** Deconvolved widefield 3D z-stack images extracted from 13 h time-lapse videos (Supplementary Videos 1, 2) of wildtype (top panels) and *Vangl2*^*Lp/+*^ (bottom panels) P3 PCLS labeled with EpCAM-FITC. **(C)** Mean net epithelial cell migration over 13 h of time-lapse imaging of P3 PCLS. *n* = 3 independent experiments using three separate mice for each group; at least three fields were quantified from each lung slice from each mouse per group per experiment. Each dot represents mean net epithelial cell migration per field. Mann–Whitney *U*-test, ***p = 0.0001. **(D)** Individual net cell migration in wildtype and *Vangl2*^*Lp/+*^ P3 PCLS after 13 h of time-lapse imaging (each dot represents a single cell). A total of 1248 cells in wildtype and 1635 cells in *Vangl2*^*Lp/+*^ P3 PCLS were tracked. Cells fall within the area highlighted in pink were highly motile and migrated between 5 and 11 μm. *n* = 3 independent experiments using three separate mice for each group. Mann–Whitney *U*-test, ***p = 0.0007. **(E)** MTT cell viability assay comparing wildtype and *Vangl2*^*Lp/+*^ P3 PCLS at the end of 13 h time-lapse; *n* = 3 independent experiments using three separate mice, with duplicate slices per condition per experiment; Mann–Whitney *U*-test. **(F)** H&E-stained sections from P3 PCLS at 0 and 72 h *ex vivo* culture of wildtype (top panels) and *Vangl2*^*Lp/+*^ (bottom panels) mice. Airspace count **(G)** and mean linear intercept (Lm) **(H)** obtained from H&E sections of P3 PCLS of wildtype and *Vangl2*^*Lp/+*^ mice at 0 and 72 h; *n* = 3 independent experiments using three separate mice, three H&E sections from each PCLS from each mouse were quantified per group per experiment. Each dot represents counts per field; one-way ANOVA with Tukey’s *post hoc* test, ***p < 0.001, *p < 0.05. All data are presented as mean ± SEM.

Disruption of *Vangl2* significantly inhibited mean net epithelial cell migration (a total linear migration of a cell from “start point” to the “end point” in 13 h) compared to WT control PCLS (1.88 vs 2.50 μm respectively; *p* = 0.0001) (Figures 1A–C and Supplementary Videos 1, 2). A large proportion of epithelial cells were relatively sessile and migrated less than 2 μm both in WT and *Vangl2*^*Lp/+*^ lung slices. However, the number of highly motile epithelial cells which migrated between 5 and 11 μm was more than double in WT PCLS compared to that of *Vangl2*^*Lp/+*^ littermates (12 vs 5%, respectively; *p* = 0.0007) (Figure 1D). MTT metabolic assays confirmed that viability was equally maintained in WT and *Vangl2*^*Lp/+*^ slices at the end of time-lapse imaging (Figure 1E).

In addition to live imaging, we compared alveologenesis in PCLS from WT and *Vangl2*^*Lp/+*^ P3 mice cultured in serum-free DMEM for 72 h. Lung slices were then embedded in paraffin, cut into 3 μm sections, and stained with hematoxylin and eosin (H&E) for imaging and subsequent morphometric analyses. Alveologenesis was compared by quantifying the number of airspaces and mean linear intercept (Lm). A smaller Lm value indicates more alveoli. In WT PCLS, the number of airspaces per square millimeter (mm^2^) area increased by 31% (171 vs 225 airspaces/mm^2^ at 0 and 72 h, respectively; *p* < 0.001), and the Lm values decreased (74.07 vs 62.84 μm at 0 and 72 h, respectively; *p* < 0.05) after 72 h of culture (Figures 1F–H), indicating that alveologenesis continued *ex vivo* (Pieretti et al., 2014; Akram et al., 2019a). By contrast, the number of airspaces and Lm values remained unaltered in *Vangl2*^*Lp/+*^ lung slices after *in vitro* culture for the same duration (178 vs 163 airspaces/mm^2^; Lm: 77.58 vs 74.49 μm at 0 and 72 h, respectively) (Figures 1F–H). MTT assays confirmed that cell viability was unaffected in WT and *Vangl2*^*Lp/+*^ PCLS after 72 h of culture (Supplementary Figure S1C). Taken together, our data show that functional deficiency of *Vangl2* perturbs alveolar epithelial cell (AEC) migration, thereby hindering postnatal alveologenesis.

### *Vangl2* Is Required for Wound Repair and Directed Cell Migration

**FIGURE 2:**
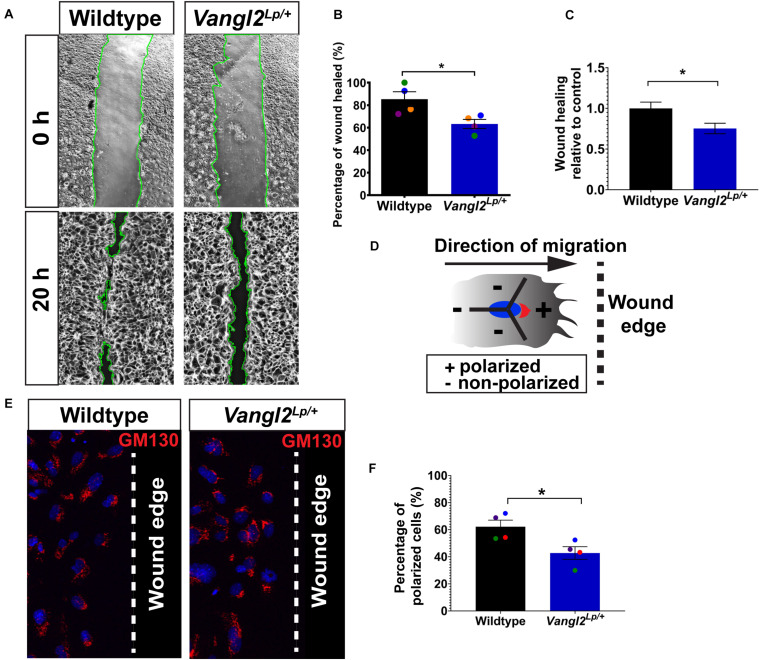
Cell polarity and wound-healing are impaired in *Vangl2*^*Lp/+*^ TECs. **(A)** Representative images showing wildtype (left panels) and *Vangl2*^*Lp/+*^ TECs (right panels) at 0 and 20 h post-scratch. Wound edges are indicated by green lines. **(B)** Percentage of wound healed measured at 20 h post-scratch in wildtype and *Vangl2*^*Lp/+*^ TECs. *n* = 4 independent experiments; TECs from three mice were pooled for each group per experiment; three technical replicates for each group per experiment; each dot represents mean percentage of wound healed per experiment. Mann–Whitney *U*-test, *p = 0.029. **(C)** Wound-healing rate in *Vangl2*^*Lp/+*^ relative to wildtype control. *n* = 4 independent experiments. Mann–Whitney *U*-test, *p = 0.029. **(D)** Schematic to illustrate polarized or non-polarized cells at leading edge as determined by the 120° arc drawn from the center of a nucleus (blue) facing the direction of migration (arrow). Golgi (red) that are located within the 120° arc (+) were considered as polarized, whereas cells with the entire or majority of the Golgi localized outside the 120° arc were classed as non-polarized. Arrow shows the direction of migration and dotted line indicates the wound edge. **(E)** Immunostaining for Golgi marker GM130 (red) in fixed wildtype and *Vangl2*^*Lp/+*^ TECs at 20 h post-scratch. Nuclei were labeled with DAPI (blue). Dotted line indicates the wound edge. **(F)** Percentage of polarized cells at 20 h post-scratch in wildtype and *Vangl2*^*Lp/+*^ TECs. *n* = 4 independent experiments; TECs from three mice were pooled for each group per experiment; three technical replicates for each group per experiment; each dot represents mean percentage of wound healed per experiment. Mann–Whitney *U*-test, *p < 0.05. All data are presented as mean ± SEM.

Next we investigated cell migration and repair capacity in *Vangl2*^*Lp/+*^ primary lung epithelial cells. We isolated tracheal epithelial cells (TECs) from WT and *Vangl2*^*Lp/+*^ mice and cultured them to confluence before inflicting a scratch wound in the cell monolayers and comparing the wound-healing capacity in WT and *Vangl2*^*Lp/+*^ TECs. The percentage of wound area healed was reduced by 20% in *Vangl2*^*Lp/+*^ TECs (63.24% in *Vangl2*^*Lp/+*^ vs 85.36% in WT; *p* = 0.029) (Figures 2A,B) or (0.75 vs 1.00 *Vangl2*^*Lp/+*^ relative to WT TECs; *p* = 0.029) (Figures 2A,C).

Given the role of the PCP pathway in establishing cell polarity during DCM (Amano et al., 1996), we then assessed whether impaired cell migration in *Vangl2*^*Lp/+*^ TECs may be attributed to aberrant cell polarity at the leading edge of a scratch wound. Cells were fixed at 20 h post-scratch and immunostained with Golgi marker GM130 to distinguish polarized and non-polarized cells as previously described (Etienne-Manneville and Hall, 2001; Cory, 2011; Poobalasingam et al., 2017). Interestingly, Golgi apparatus appeared randomly localized in *Vangl2*^*Lp/+*^ TECs at the leading edge compared with leading WT TECs, which showed a higher proportion of cells with the Golgi facing the direction of migration (Figure 2E). Quantitative analysis of GM130 position demonstrated that around 62% of the WT TECs at the leading edge were polarized compared with only 44% in *Vangl2*^*Lp/+*^ TECs (*p* = 0.029) (Figure 2F). These data confirmed that in primary lung epithelial cells, VANGL2 plays a role in wound repair following injury by regulating cell polarity during DCM.

### VANGL2 Dysfunction Leads to Highly Disrupted Actin Cytoskeleton and Focal Adhesions

**FIGURE 3:**
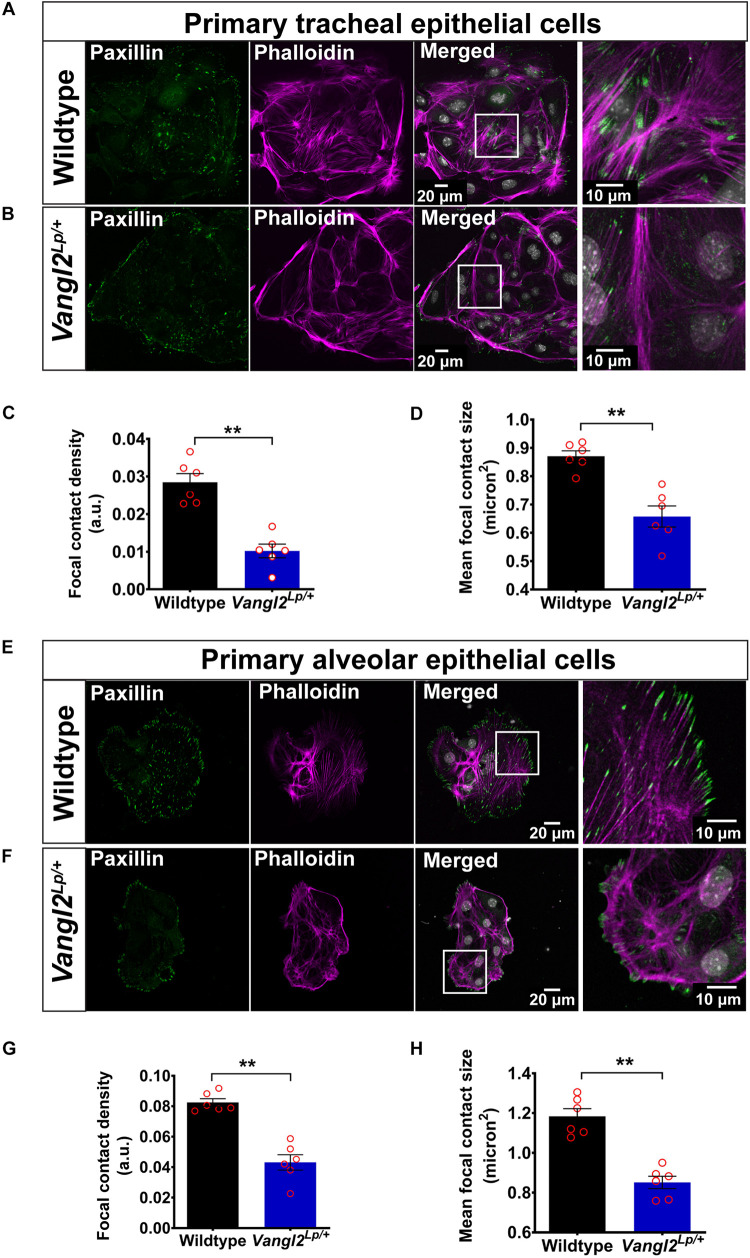
VANGL2 dysfunction leads to highly disrupted actin cytoskeleton and focal adhesions. Representative images showing wildtype **(A)** and *Vangl2*^*Lp/+*^ **(B)** TECs labeled with phalloidin (magenta) to visualize F-actin and paxillin (green) to stain focal contacts. Nuclei were stained with DAPI (gray). Quantification of focal contact density **(C)** and mean focal contact size (micron^2^) **(D)** in wildtype and *Vangl2*^*Lp/+*^ TECs. *n* = 3 independent experiments; TECs from three mice were pooled for each group per experiment; two technical replicates for each group per experiment; images from at least three fields were taken per technical replicate; each dot represents mean value per technical replicate. Mann–Whitney *U*-test, **p < 0.01. Representative images showing wildtype **(E)** and *Vangl2*^*Lp/+*^ **(F)** AECs labeled with phalloidin (magenta) and paxillin (green). Nuclei were labeled with DAPI (gray). Quantification of focal contact density **(G)** and mean focal contact size (micron^2^) **(H)** in wildtype and *Vangl2*^*Lp/+*^ AECs. *n* = 3 independent experiments; AECs from one mouse per group for each experiment; two technical replicates for each group per experiment; at least three fields were taken per technical replicate; each dot represents mean value per technical replicate. Mann–Whitney *U*-test, **p < 0.01. All data are presented as mean ± SEM.

To investigate whether impaired cell mechanics were associated with the cell migration defects in *Vangl2*^*Lp/+*^ lungs, we first investigated actin cytoskeleton organization and FA integrity in adult WT and *Vangl2*^*Lp/+*^ epithelial cells. Phalloidin stained stress fibers throughout WT TECs (Figure 3A). In contrast, *Vangl2*^*Lp/+*^ TECs exhibited diffuse and highly disrupted actin filaments that were sparsely distributed and barely visible (Figure 3B). Cortical actin was visible in both WT and *Vangl2*^*Lp/+*^ TECs. These microscopic changes in actin distribution within adult *Vangl2*^*Lp/+*^ epithelial cells are consistent with our previous findings of aberrant actin distribution and regulation in whole embryonic homozygous and heterozygous *Vangl2*^*Lp*^ lung tissues (Poobalasingam et al., 2017).

Immunostaining for the FA protein, paxillin, in WT TECs demonstrated a high number of elongated FAs at the tips of stress fibers and small dot-like FAs at the cell periphery (Figure 3A). This was strikingly different from *Vangl2*^*Lp/+*^ TECs, in which FAs were markedly reduced and large FAs capping stress fibers were rarely present (Figure 3B). Quantification of both mean FA size and FA density (total FA area normalized to total cell area) revealed a substantial reduction in *Vangl2*^*Lp/+*^ TECs (*p* < 0.01) (Figures 3C,D). Similarly, compared to WT cells (Figure 3E), AECs isolated from *Vangl2*^*Lp/+*^ mutant mice also exhibited highly disordered actin cytoskeleton (Figure 3F) and a dramatic reduction in both mean FA size and density (*p* < 0.01) (Figures 3G,H). Immunostaining with pan-cytokeratin, an epithelial cell marker, was performed for TECs and AECs in each experiment to confirm the purity of epithelial cell populations (Supplementary Figure S2).

**FIGURE 4:**
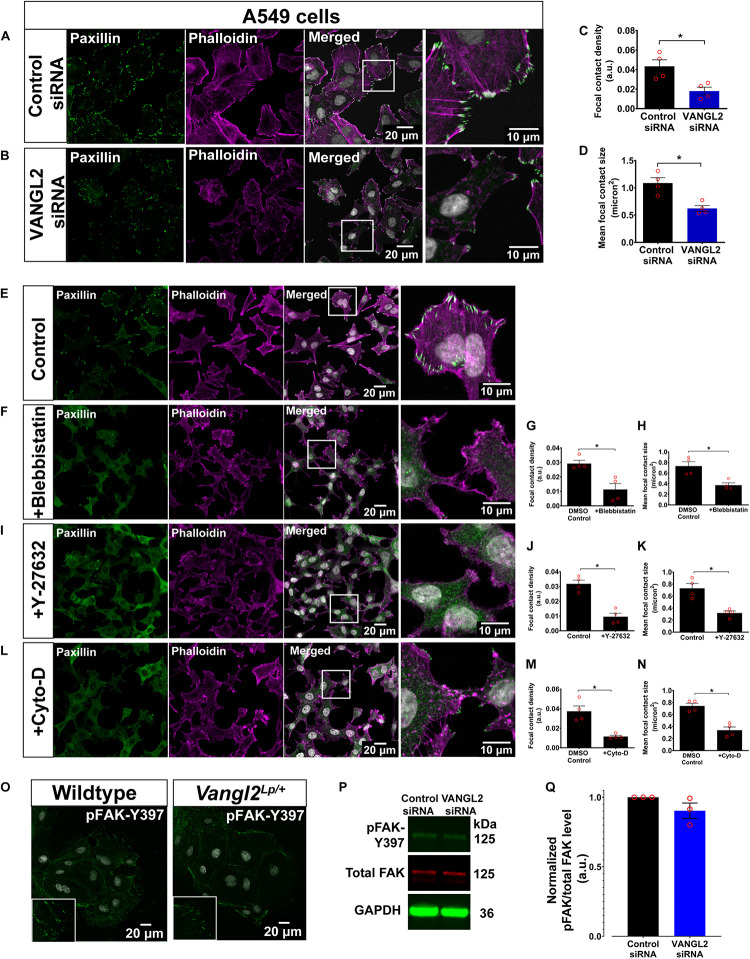
A549 treated with actomyosin inhibitors phenocopy actin cytoskeleton and focal adhesion aberrations following siRNA knockdown of *VANGL2*. Representative images showing A549 transfected with **(A)** control siRNA or **(B)** *VANGL2* siRNA. A549 labeled with phalloidin (magenta) to visualize F-actin and paxillin (green) for focal contacts. Nuclei were stained with DAPI (gray). Quantification of focal contact density **(C)** and mean focal contact size (micron^2^) **(D)** in control siRNA- or *VANGL2* siRNA-treated A549 cells. *n* = 4 independent experiments; two technical replicates for each group per experiment; each dot represents mean value per experiment. Mann–Whitney *U*-test, ^∗^*p* < 0.05. Representative images showing control A549 without treatment **(E)**, A549 treated with 25 μM blebbistastin for 30 min **(F)**, 10 μM Y-27632 for 1 h **(I)**, or 0.5 μM cytochalasin D for 30 min **(L)**, and their corresponding focal contact density and mean focal contact size quantification **(G,H,J,K,M,N)**. No morphological difference was observed in the DMEM-DMSO control or DMEM only control cells so only representative control cell images are shown. Cells were stained with phalloidin (magenta) and paxillin (green). Nuclei were stained with DAPI (gray). **(O)** Representative images showing wildtype and *Vangl2*^*Lp/+*^ TECs stained with pFAK-Y397 (green). Nuclei were stained with DAPI (gray). Representative western blots show the levels of pFAK-Y397 (green), total FAK (red), reference protein GAPDH (green) **(P)**, and quantification of pFAK levels normalized to total FAK levels **(Q)**. See Supplementary Figure S4 for whole western blot. *n* = 3 independent experiments. All data are presented as mean ± SEM.

To further validate our findings, we conducted *VANGL2* knockdown by siRNA transfection in human AECs (A549). The siRNA knockdown efficiency was assessed by qRT-PCR, as shown in Supplementary Figure S3A. In agreement with the findings described above, *VANGL2*-depleted A549 cells recapitulated overt anomalies in both actin cytoskeleton and FAs (Figures 4A–D). These morphological aberrations resembled A549 cells treated with different actomyosin inhibitors: para-nitroblebbistatin (blebbistatin; an inhibitor of non-muscle myosin II ATPase activity) (Képiró et al., 2014), ROCK inhibitor, Y-27632 (Uehata et al., 1997), and actin polymerization inhibitor, cytochalasin D (Schliwa, 1982) (Figures 4E–N). Scratch assays conducted in A549 cells treated with blebbistatin or cytochalasin D also showed significantly impaired cell migration (Supplementary Figures S3B–E) as shown in primary *Vangl2*^*Lp*^ TECs (Figure 2). Altogether, these results suggest that disrupted FAs and actin cytoskeleton in *VANGL2*-deficient cells contribute to impaired cell migration.

Since FAK is known to be one of the major kinases that regulate the formation and maturation of FAs (Hamadi et al., 2005), we next investigated whether phosphorylated FAK (pFAK; active form) is affected in *Vangl2*^*Lp/+*^ TECs. Immunostaining for pFAK-Y397 revealed an altered pFAK pattern in *Vangl2*^*Lp/+*^ TECs (Figure 4O). However, it was technically challenging to obtain sufficient materials from primary cells to quantify pFAK levels by western blotting, therefore, given the similar phenotype observed in both *Vangl2*^*Lp/+*^ primary cells and *VANGL2*-depleted A549s, lysates from control- or *VANGL2*-siRNA-treated A549 cells were used to quantify pFAK and total FAK levels. Our results showed a slight reduction in normalized pFAK levels following *VANGL2*-siRNA knockdown. However, the difference was not significant (*p* = 0.16) (Figures 4P,Q and Supplementary Figure S4).

### VANGL2 Disruption Inhibits Phospho-MLC2-Mediated Traction Force Generation

**FIGURE 5:**
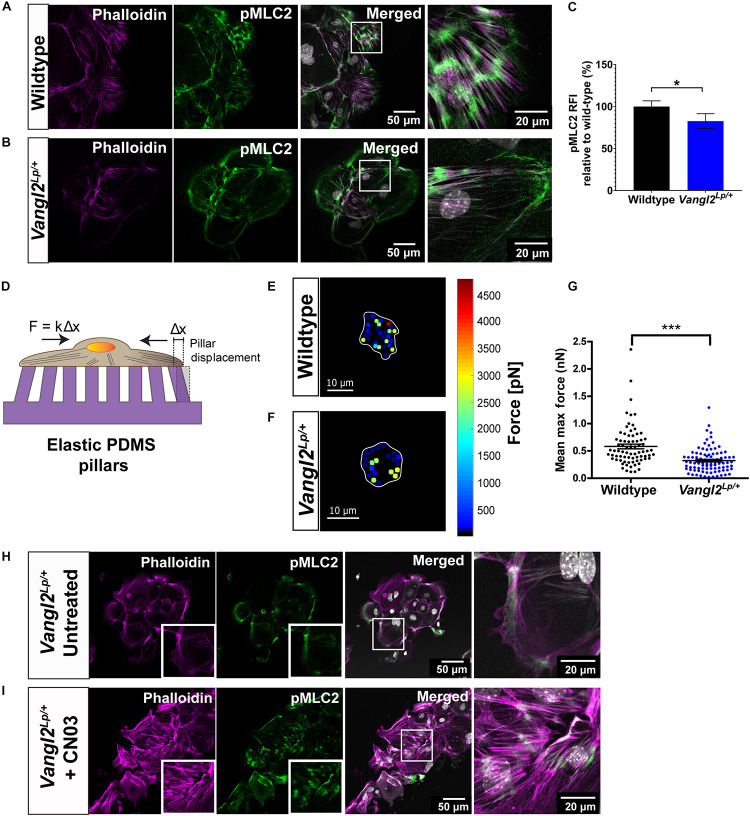
VANGL2 regulates MLC2 activation and force generation via RhoA signaling. Representative images showing wildtype **(A)** and *Vangl2*^*Lp/+*^ **(B)** TECs stained with pMLC2 (green) and phalloidin (magenta). Nuclei are shown in gray (DAPI). **(C)** Quantification of pMLC2 relative fluorescence intensity (RFI) in *Vangl2*^*Lp/+*^ relative to wildtype (presented as mean ± SEM). *n* = 3 independent experiments; TECs from three mice were pooled for each group per experiment; two technical replicates for each group per experiment; at least three fields were taken per technical replicate. Mann–Whitney *U*-test, *p < 0.05. **(D)** Schematic representation of the elastic micropillars used to measure cellular traction forces. Cells were seeded on a rat-tail collagen I coated array of micropillars and incubated at 37°C with 5% CO_2_ for 24 h. Individual pillar deflection from its original position in the array (Δx) is tracked via time-lapse microscopy and converted to traction force based on the pillar spring constant (*k* = 1.36 nN/μm). Heat maps of the traction forces distribution in a representative wildtype **(E)** and *Vangl2*^*Lp/+*^ **(F)** TEC. Cell boundary is outlined in white. **(G)** Quantification of the mean maximum traction forces exerted by wildtype (*n* = 75 cells) and *Vangl2*^*Lp/+*^ TECs (*n* = 92 cells) on the micropillars (presented as mean ± SEM). *n* = 2 independent experiments; in each experiment, tracheas from three separate mice were pooled for each group; two technical replicates for each group per experiment; each dot represents mean maximum traction force per cell. Two-tailed unpaired Student’s *t*-test, ***p < 0.001. Representative images showing *Vangl2*^*Lp/+*^ TECs without treatment **(H)** and *Vangl2*^*Lp/+*^ TECs treated with Rho activator, CN03 **(I)**. TECs were stained with pMLC2 (green) and phalloidin (magenta). Nuclei were stained with DAPI (gray). *n* = 3 independent experiments; TECs from three mice were pooled for each group per experiment; two technical replicates for each group per experiment.

Phosphorylated MLC2 (pMLC2) induces actomyosin contractility and enables cells to generate forces required for cell adhesion and cell motility (Amano et al., 1996; Pandya et al., 2017). Thus, we next interrogated whether MLC2 activation is affected in *Vangl2*^*Lp/+*^ TECs. Dual staining of pMLC2 and phalloidin demonstrated high levels of pMLC2 intermittently distributed along the F-actin bundles in WT TECs (Figure 5A). In contrast, a marked reduction in pMLC2 was observed in *Vangl2*^*Lp/+*^ TECs, with an overall decreased formation of actomyosin bundles (Figure 5B). Relative fluorescence intensity (RFI) analysis revealed a significant decrease in pMLC2 in *Vangl2*^*Lp/+*^ TECs compared with their WT counterparts (*p* = 0.029) (Figure 5C).

To determine whether the abnormal actomyosin organization may affect the ability of *Vangl2*^*Lp/+*^ TECs to exert forces on substrates, we measured traction forces applied by TECs, using elastic micropillars. TECs were seeded on an array of elastic polydimethylsiloxane (PDMS) micropillars coated with rat-tail collagen I and the deflection of each pillar, which was proportional to cellular traction force was assessed (Figure 5D). Our results showed that traction forces were predominantly distributed around the cell periphery, in both WT and *Vangl2*^*Lp/+*^ TECs (Figures 5E,F). Quantitative analysis of the mean maximum forces exerted on the pillars indicated that *Vangl2*^*Lp/+*^ TECs generated significantly less traction forces (mean: 322 pN, median: 276 pN) compared with WT TECs (mean: 582 pN, median: 487 pN) (*p* < 0.001) (Figure 5G). These data demonstrate that functional VANGL2 is required for the formation of optimal actomyosin networks that drive traction force generation.

### Rho Activator Restores Actomyosin Organization in *Vangl2*^*Lp/+*^

Planar cell polarity is known to regulate actin cytoskeleton remodeling through its downstream effector RhoA signaling (Munoz-Soriano et al., 2012; Davey and Moens, 2017; Henderson et al., 2018). Thus, we next investigated whether exogenous activation of RhoA signaling could rescue the actomyosin defects in adult *Vangl2^*Lp/+*^. Vangl2^*Lp/+*^* TECs were treated with Rho activator II, CN03, for 3 h. Immediately after treatment, cells were fixed and labeled with phalloidin and anti-pMLC2 antibody. Untreated *Vangl2*^*Lp/+*^ TECs were used as a control (Figure 5H). Confocal images showed that CN03 treatment led to striking changes in actomyosin organization in *Vangl2*^*Lp/+*^ TECs. CN03 treatment markedly increased the formation of stress fibers and pMLC2 expression was considerably enhanced (Figure 5I), demonstrating that actomyosin defects caused by VANGL2 disruption can be ameliorated by exogenously activating RhoA signaling.

### Reduced YAP Signaling in *Vangl2*^*Lp*^ Mutant Lungs

**FIGURE 6:**
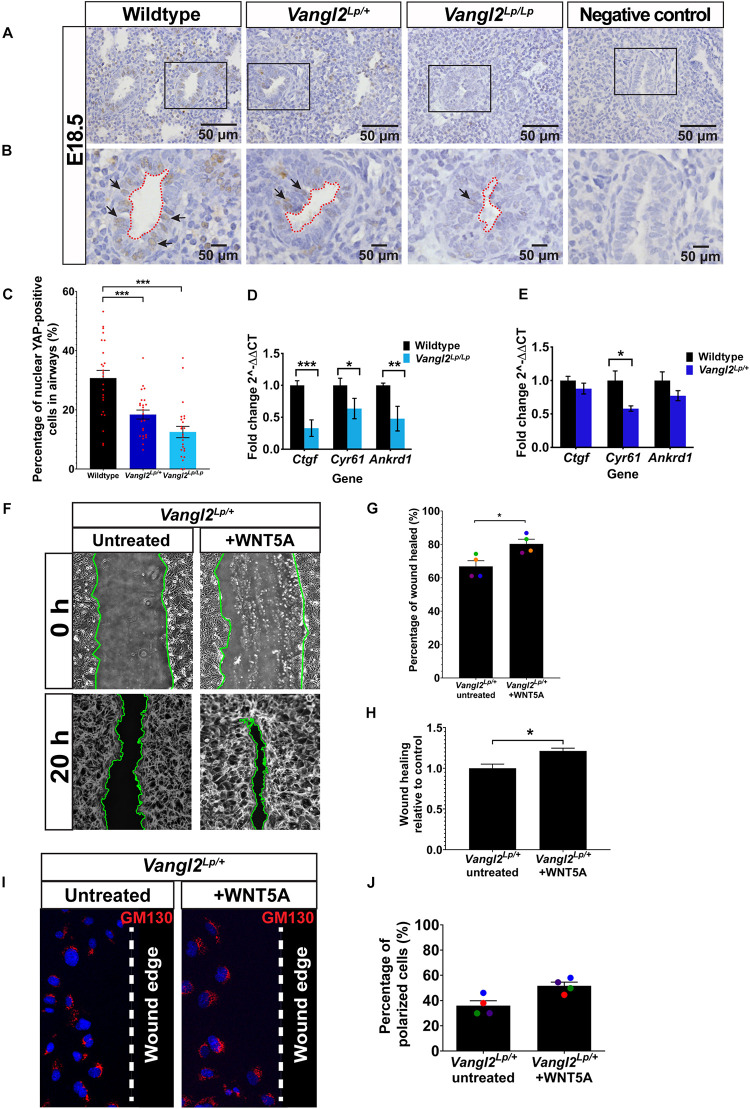
Reduced YAP signaling in embryonic *Vangl2*^*Lp*^ airways and WNT5A rescues cell migration defects in *Vangl2*^*Lp/+*^. **(A,B)** Representative images show immunostaining for YAP in E18.5 wildtype, heterozygous *Vangl2*^*Lp/+*^, and homozygous *Vangl2*^*Lp/Lp*^ mouse lungs. Sections were counterstained with hematoxylin. Negative controls with primary antibody omitted are shown. Insets **(B)** show the morphology of airway lumen. Arrows indicate nuclear YAP staining. **(C)** Percentage of nuclear YAP-positive cells in the airways. *n* = 3 mice per group; eight airways were quantified per mouse; a total of 1389 cells (wildtype), 1381 cells (*Vangl2*^*Lp/+*^), and 995 cells (*Vangl2*^*Lp/Lp*^) were analyzed (presented as mean ± SEM); one-way ANOVA with Tukey’s *post hoc* test, ***p < 0.001. Histograms show transcript levels for YAP target genes: *Ctgf*, *Cyr61*, and *Ankrd1* in E18.5 wildtype vs *Vangl2*^*Lp/Lp*^ **(D)** and adult wildtype vs *Vangl2*^*Lp/+*^ lungs **(E)**. *n* = 3 independent experiments; three mice per genotype; each experiment was run in triplicate. Data are presented as mean ± SEM; Mann–Whitney *U-*test, *p < 0.05, **p < 0.01, ***p < 0.001. **(F)** Representative images showing untreated and WNT5A-treated *Vangl2*^*Lp/+*^ TECs at 0 and 20 h post-scratch. Wound edges are indicated by green lines. Percentage of wound healed 20 h after wound scratch in untreated *Vangl2*^*Lp/+*^ TECs and *Vangl2*^*Lp/+*^ TECs treated with WNT5A **(G)** and wound-healing rate in WNT5A-treated *Vangl2*^*Lp/+*^ TECs in relative to untreated *Vangl2*^*Lp/+*^ TEC controls **(H)**. Data are presented as mean ± SEM. *n* = 4 independent experiments; TECs from three mice were pooled for each group per experiment; three technical replicates for each group per experiment; each dot represents mean percentage of wound healed per experiment. Mann–Whitney *U*-test, *p < 0.05. **(I)** Immunostaining for Golgi marker (GM130; red) in fixed untreated and WNT5A-treated *Vangl2*^*Lp/+*^ TECs 20 h post-scratch to determine cell polarity at leading edge. Nuclei were labeled with DAPI (blue) and dotted line indicates the wound edge. **(J)** Percentage of polarized cells 20 h post-scratch in untreated and WNT5A-treated *Vangl2*^*Lp/+*^ TECs (presented as mean ± SEM). *n* = 4 independent experiments; TECs from three mice were pooled for each group per experiment; three technical replicates for each group per experiment. Mann–Whitney *U*-test.

Nuclear YAP is used as a read-out of active mechanosignaling and recent data have shown that YAP deficiency has profound effects on lung development (Lin et al., 2017). In order for cells to process the mechanical signals, intracellular mechanotransducers, such as YAP, that can detect and respond to mechanical cues play an essential role. To determine whether YAP signaling is affected in *Vangl2*^*Lp*^ lungs, we next performed immunohistochemistry of YAP on embryonic E18.5 WT, heterozygous, and homozygous *Vangl2*^*Lp*^ lung sections and compared the percentage of airway epithelial cells that were positive for nuclear (active) YAP. Interestingly, as shown in Figures 6A–C, the percentage of cells with nuclear YAP was significantly decreased in the airways of *Vangl2*^*Lp/+*^ (18.38%; *p* < 0.001) and *Vangl2*^*Lp/Lp*^ (11.97%; *p* < 0.001) lungs compared with WT control (30.71%). To determine whether reduced active nuclear YAP is also associated with increased cytoplasmic sequestration of non-active, phospho-YAP in *Vangl2*^*Lp*^ lung sections, immunofluorescence of pYAP (Ser127) was performed on embryonic E18.5 WT, heterozygous, and homozygous *Vangl2*^*Lp*^ lung cryosections. RFI analysis revealed a significant increase in cytoplasmic pYAP in the airways of *Vangl2*^*Lp/Lp*^ lungs compared with WT control (*p* < 0.05) (Supplementary Figure S5). RFI of pYAP was also higher in *Vangl2*^*Lp*^ heterozygous lungs, though this did not reach statistical significance (Supplementary Figure S5). Consistent with our previous findings (Yates et al., 2010b; Poobalasingam et al., 2017), lung architectural defects were also observed in *Vang2*^*Lp/+*^ and *Vang2*^*Lp/Lp*^ lungs. Nuclear YAP was also present in a proportion of mesenchymal cells. However, due to the altered architecture in *Vangl2*^*Lp*^ lungs, this was difficult to quantify and therefore restricted our quantification to the airways only.

As well as examining YAP at the protein level, we assessed the transcript levels of known YAP target genes: *Ctgf* (connective tissue growth factor) (Zhao et al., 2008), *Cyr61* (cysteine rich angiogenic inducer 61) (Choi et al., 2015), and *Ankrd1* (ankyrin repeat domain-containing protein 1) (Choi et al., 2015) by qRT-PCR, using total RNA extracted from E18.5 and adult WT and *Vangl2*^*Lp*^ mutant lungs. In agreement with our immunostaining data, qRT-PCR results showed a significant reduction in transcript levels of *Ctgf* (*p* < 0.001), *Cyr61* (*p* < 0.05), and *Ankrd1* (*p* < 0.01) in embryonic (E18.5) *Vangl2*^*Lp/Lp*^ lungs (Figure 6D). However, in adult *Vangl2*^*Lp/+*^ lungs, where only heterozygous tissue was available due to neonatal lethality of homozygotes, only *Cyr61* transcript levels were significantly decreased compared to WT littermate lungs (*p* < 0.05) (Figure 6E). There was a slight reduction in *Ctgf* and *Ankrd1* levels but the difference in these genes was not statistically significant in the adult (Figure 6E). These results suggest that YAP signaling is dysfunctional in *Vangl2*^*Lp*^ mutant lungs.

### WNT5A Rescues Cell Migration Defects in *Vangl2*^*Lp/+*^ TECs

WNT5A is a known ligand for the PCP pathway (Qian et al., 2007; Komiya and Habas, 2008; Witze et al., 2008) and plays a key role in both alveologenesis and the response to lung injury (Poobalasingam et al., 2017; Nabhan et al., 2018; Li et al., 2020). However, it is still unclear which downstream Wnt pathway(s) respond to WNT5A following injury to facilitate repair.

Thus, we tested whether addition of exogenous WNT5A would improve cell migration in *Vangl2*^*Lp/+*^ TECs. Interestingly, scratch assay results demonstrated that treatment with WNT5A consistently increased the percentage of wound area healed (80.3%) compared with untreated *Vangl2*^*Lp/+*^ TECs (66.8%) (Figures 6F,G). Relative wound-healing rate showed a 1.21-fold increase in WNT5A-treated *Vangl2*^*Lp/+*^ TECs (*p* = 0.0434) (Figure 6H). Next, we tested whether the effect of WNT5A on cell migration was due to restoration of cell polarity. Analysis of the GM130 staining revealed that WNT5A treatment increased the percentage of polarized cells at the leading edge (54.38% cells were polarized) compared with 35.94% in untreated *Vangl2*^*Lp/+*^ TECs. However, the difference was not statistically significant (*p* = 0.0571) (Figures 6I,J).

## Discussion

Our previous work has shown that the PCP pathway is important for development, homeostasis, and repair of the lungs, highlighting the fundamental role of this pathway in lung biology (Yates et al., 2010b, 2013; Poobalasingam et al., 2017). In various tissues, planar polarity is regulated by molecular gradients of key molecules, for example, Frizzled, or by physical forces, for example, the anisotropic forces that regulate PCP and wing blade shaping in *Drosophila* (Gao et al., 2011; Aw et al., 2016; Henderson et al., 2018). Mechanical forces play an essential role in branching morphogenesis during lung development, helping to shape the airway epithelium (Kim and Nelson, 2012; Varner et al., 2015). Similarly, cyclic mechanical strain associated with mechanical or spontaneous ventilation is a key determinant of survival in patients with injured lungs. Moreover, dysregulation of mechanical forces can have detrimental effects on lung function leading to tissue injury and disease (Kim and Nelson, 2012; Waters et al., 2012; Varner et al., 2015).

Mechanobiology is concerned with how cells sense and translate forces to regulate cell behaviors like morphogenesis and migration by converting mechanical stimuli into biochemical signals through mechanoregulators. Both mechanosignaling and PCP signaling result in modulation of the actin cytoskeleton but how these two pathways are connected at the molecular level is not understood. A growing number of studies have begun to discern the importance of mechanoregulators like YAP in lung development and repair. In particular, YAP is critical for lung branching morphogenesis *in utero* while TAZ deletion affects post-natal alveologenesis leading to an emphysema-like phenotype in adult mice (van Soldt et al., 2019; Isago et al., 2020). YAP and TAZ also regulate regeneration of the adult mouse lungs in response to *Streptococcus pneumoniae*-mediated injury (Lacanna et al., 2019). Here we have shown that mechanoregulation of the planar polarity pathway via *Vangl2* is integral to PCP driven cell behaviors such as DCM and morphogenesis.

Our previous data showed that perturbed VANGL2 function in *Vangl2*^*Lp/+*^ mice leads to disorganization of the actin cytoskeleton, resulting in alveologenesis defects (Poobalasingam et al., 2017). Zhang et al. (2020) recently reported similar alveologenesis defects in different *Vangl2*^*f/f*^ conditional mouse mutants, further supporting the role for PCP pathway in lung development. In this study, we used live imaging to show conclusively that VANGL2 has an important role in cell migration. Reduced movement of AECs and impaired alveologenesis was evident in *Vangl2*^*Lp/+*^ PCLS compared to WT. To dissect the cellular mechanisms underlying this reduced cell migration, we used primary TECs and AECs isolated from adult WT and *Vangl2*^*Lp/+*^ mice, as well as siRNA intervention in A549 cells. We demonstrated using *in vitro* wound-healing assays that *Vangl2*^*Lp/+*^ TECs exhibited reduced DCM. In *Vangl2*^*Lp/+*^ TEC monolayers, cells at the leading edge of the wound did not display coordinated polarity. In migrating cells, crosstalk between PCP proteins and cytoskeletal components is required for polarized organization of the cytoskeleton that underlies front-rear polarity (Raman et al., 2018). The *Vangl2*^*Lp*^ mutation is known to affect trafficking of VANGL2 protein to the membrane (Gao et al., 2011; Seo et al., 2017) and we have previously shown a reduction in β-catenin at the cell membranes in *Vangl2*^*Lp/+*^ lungs (Poobalasingam et al., 2017). Membrane associated β-catenin is important to maintain proper actin cytoskeleton structure and cell tension (Abreu-Blanco et al., 2012), together with the data shown in this manuscript our findings indicate that VANGL2 dysfunction disrupts the actin cytoskeleton and thereby affects the polarization of the cytoskeleton required for DCM.

Mechanosensing, the process by which cells assess their environment to respond to mechanical cues, relies on the formation of FAs, through which cells can apply force generated by actomyosin-mediated contractility (Evans and Calderwood, 2007; Geiger et al., 2009). Our results demonstrated that VANGL2 regulates FA size and density, a phenotype that is likely to affect mechanosignaling. To measure the traction forces generated by *Vangl2*^*Lp/+*^ cells as a functional readout of the underlying cytoskeletal defects leading to the FA changes in *Vangl2*^*Lp/+*^ cells, we employed micropillar arrays. These arrays are composed of uniformly spaced needle-like microposts of user-defined rigidity that allow discrete attachment of a cell. This enables sensitive quantification of traction force by measuring the deflection of pillars, using a simple mathematical model as shown in Figure 5D (Yang et al., 2011; Chronopoulos et al., 2016). We demonstrated significantly reduced cellular traction force when VANGL2 function is disrupted.

We also showed that addition of a Rho activator restored MLC2 activation and actomyosin organization in *Vangl2*^*Lp/+*^ cells, confirming that VANGL2 regulates actomyosin contractility via its effector RhoA. This is in line with previous work showing inhibition of ROCK (a downstream effector of RhoA) by selective inhibitor Y-27632 leads to reduced MLC2 phosphorylation (Unbekandt et al., 2014). Altogether, our findings demonstrate a direct role for VANGL2, a core PCP component, in the regulation of traction force generation for the first time. The correlation between FA size and magnitude of force exerted at the FA has been a longstanding debate, previous studies having shown either a positive linear (Balaban et al., 2001; Schwarz et al., 2002; Goffin et al., 2006) or inverse relationship (Beningo et al., 2001), as well as more complicated findings that suggest positive correlation is limited to early FA growth and is diminished in mature FAs (Stricker et al., 2011). Our findings in this study have shown a concomitant decrease in FA size and traction force in *Vangl2*^*Lp/+*^ TEC, which support a positive correlation between FA size and force applied at FA.

Park et al. (2015) previously found that YAP/TAZ can act as downstream effectors of the non-canonical/PCP pathway in mediating cell migration and osteogenic differentiation via Frizzled and ROR. Our study has discovered that *Vangl2*^*Lp*^ is associated with reduced nuclear (active) YAP and increased cytoplasmic phospho-YAP (non-active) in the lung epithelium. Since YAP provides a read-out of mechanosignaling activity (Dupont et al., 2011; Wada et al., 2011; Dobrokhotov et al., 2018), our data provide evidence that VANGL2 has an active role in mechanosignaling.

Narrow or collapsed airway lumens are a feature of genetically modified mouse mutants of the PCP pathway (Yates et al., 2010b; Poobalasingam et al., 2017) and mechanical forces are important for airway branching morphogenesis (Varner et al., 2015). Moreover, YAP deficiency, which results in defective mechanical force generation, disrupts lung branching morphogenesis in mice (Lin et al., 2017). The reduction in YAP activity detected in the airways of *Vangl2*^*Lp*^ mice suggests that the collapsed airway lumen that are a prominent feature of embryonic *Vangl2*^*Lp*^ mouse lungs could result from the perturbation of tensile forces within *Vangl2*^*Lp*^ airway epithelial cells, as a result of defective mechanosignaling.

Our findings also raise important questions for future investigation. pFAK is known to regulate the formation of stress fibers and FAs (Schiller and Fässler, 2013; Oakes and Gardel, 2014; Sun et al., 2016). Immunostaining for pFAK did not reveal overt changes in pFAK distribution or pattern in heterozygous *Vangl2*^*Lp/+*^ TECs, suggesting that the loss of pFAK may not provide a significant contribution to the highly disrupted cytoskeletal organization and FA structures in *Vangl2*^*Lp/+*^ cells. It is also possible that homozygous *Vangl2*^*Lp/Lp*^ cells would exhibit more discernible differences in the pFAK distribution and protein level compared to their heterozygous counterparts. However, since *Vangl2*^*Lp/Lp*^ mice are not viable after birth, we could not assess TECs from homozygous mice.

WNT5A is most frequently regarded as a non-canonical Wnt ligand that regulates the PCP pathway (Qian et al., 2007; Komiya and Habas, 2008; Witze et al., 2008). In the lungs, WNT5A has emerged as an important mediator of alveologenesis and repair following injury (Poobalasingam et al., 2017; Nabhan et al., 2018; Li et al., 2020). While evidence shows that VANGL2 has a central role in WNT5A-mediated PCP signaling (Babayeva et al., 2011; Poobalasingam et al., 2017; Yang et al., 2017; Rao-Bhatia et al., 2020), surprisingly, we demonstrated that WNT5A improved cell migration in *Vangl2*^*Lp/+*^ cells. These findings indicate that WNT5A can still induce cell migration when VANGL2 function is perturbed. There are a number of possibilities as to how this could transpire. For example, it is possible that in the absence of an intact PCP pathway, WNT5A signals via the alternative canonical Wnt/β-catenin pathway in *Vangl2*^*Lp*^. In some contexts, canonical Wnt signaling is increased in response to disrupted PCP signaling (Weidinger and Moon, 2003; Westfall et al., 2003). However, we did not find any change in canonical Wnt signaling in adult *Vangl2*^*Lp/+*^ lungs (Poobalasingam et al., 2017). It is also possible that in *Vangl2*^*Lp/+*^ lungs, some functional VANGL2 protein remains and this is sufficient to transduce WNT5A to promote wound-healing. Studies have shown that WNT5A can bind to a number of different receptors to mediate PCP signaling in diverse cell contexts. One prominent receptor that WNT5A binds to mediate actin cytoskeleton remodeling via the PCP pathway is ROR2 (Nishita et al., 2010; Gao et al., 2011). In particular, ROR2 has an important role in the phosphorylation of VANGL2 in response to WNT5A (Gao et al., 2011). Recently, two separate studies reported that an axis of WNT5A-ROR (Li et al., 2020) or WNT5A-ROR2-VANGL2 (Zhang et al., 2020) is required for alveologenesis, based on the similar phenotypes observed in a number of different conditional mouse mutants of these genes. Intriguingly, WNT5A has been shown to bind to ROR2 to mediate cell invasion independently of the PCP pathway (Enomoto et al., 2009) and ROR1/ROR2 can act as direct receptors for WNT5A to recruit guanine exchange factors (GEFs) and activate Rac1 and RhoA (Yu et al., 2016), supporting the idea that WNT5A can signal through more than one route. In addition to ROR2, RYK is another receptor that binds to WNT5A and VANGL2 to regulate PCP signaling (Andre et al., 2012; Duan et al., 2017). It remains unclear which is the predominant WNT5A receptor that mediates PCP signaling in *Vangl2*^*Lp*^ lungs and further studies will be required to comprehensively address this.

WNT5A is a current target of interest for treatment of several lung diseases in which there is aberrant expression of this ligand including: chronic obstructive pulmonary disease (COPD)/emphysema, idiopathic pulmonary arterial hypertension (IPAH), bronchopulmonary dysplasia (BPD), and interstitial pneumonia (UIP) (Vuga et al., 2009; Baarsma et al., 2017; Yuan et al., 2019; Li et al., 2020; Sucre et al., 2020; Vladar and Königshoff, 2020). In addition, WNT5A has an important role in progenitor cell induction following lung injury (Nabhan et al., 2018). It is also notable that several independent datasets from emphysema patients, in which repair capacity is diminished, show significant downregulation of *VANGL2* and *WNT5A* (Ezzie et al., 2012; Poobalasingam et al., 2017; Zhang et al., 2020). However, contradictory findings with regard to pro-repair/migratory (Witze et al., 2008; Vuga et al., 2009; Gao et al., 2011; Poobalasingam et al., 2017; Yuan et al., 2019; Li et al., 2020; Zhang et al., 2020) or anti-repair effects (Baarsma et al., 2017; Wu et al., 2019; Sucre et al., 2020) of WNT5A suggest that the complex mechanism of action of WNT5A may be cell type or tissue context-dependent or may differ depending on the balance between PCP and other signaling pathways, such as canonical Wnt signaling. Thus, it will be important to carry out further detailed investigation of how WNT5A stimulates repair in *Vangl2*^*Lp/+*^ mice.

**FIGURE 7:**
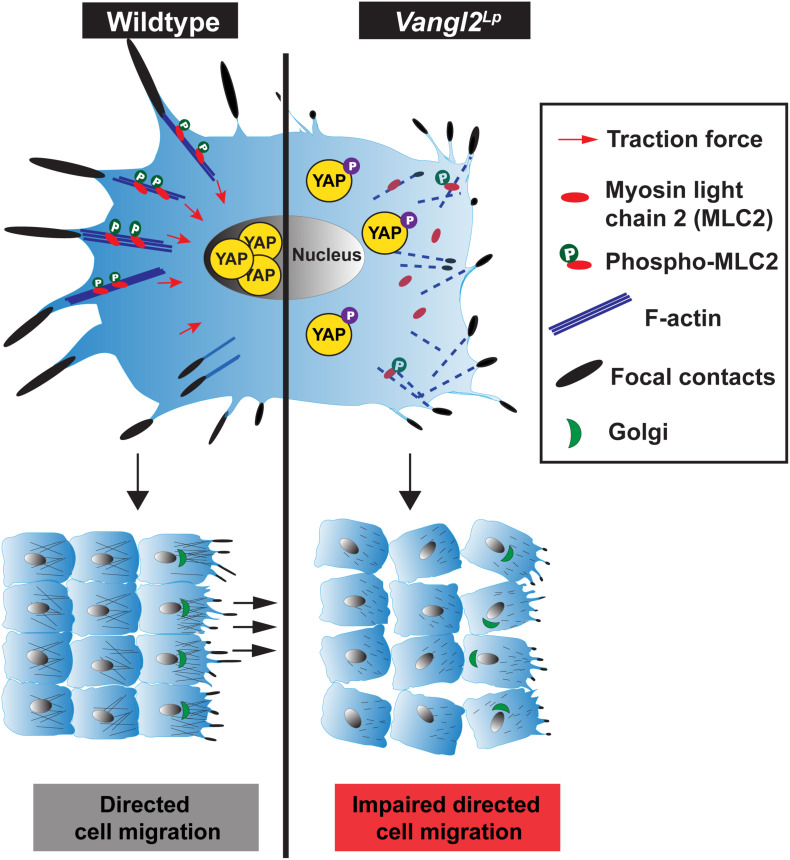
Comparison of cellular mechanics in wildtype and *Vangl2*^*Lp*^ cells. (Left) In a normal cell, VANGL2 regulates the activation of myosin light chain 2 (MLC2) and F-actin and induces intracellular contractility. This gives rise to the generation of cellular traction force (red arrows) and promotes the formation of focal adhesion complexes. High mechanical cues within a cell then activate YAP signaling, rendering the translocation of YAP to the nucleus, leading to the transcriptional induction of YAP target genes. The optimal coordination of focal complexes, actomyosin contractile activity at the rear and pushing force at the front, as well as VANGL2-regulated cell polarity enables directed cell migration. (Right) In a *Vangl2*^*Lp*^ cell, VANGL2 dysfunction leads to highly disrupted actin cytoskeleton, aberrant focal complex formation, and inhibition of MLC2 activation. This causes defective actomyosin-mediated contractility, which hampers traction force generation. Attenuated mechanosignaling within the cell inhibits activation of mechanosensitive protein YAP. YAP is phosphorylated, rendering its sequestration in the cytoplasm, thereby causing the transcriptional suppression of YAP target genes. Perturbation of traction force generation, aberrant focal contact formation, the lack of pushing force caused by highly disrupted actin cytoskeleton, and defective VANGL2-regulated cell polarity impede directed cell migration.

Our study shows that beyond establishing tissue patterning, VANGL2, a core PCP component, plays a pivotal role in the regulation of cell mechanobiology. We provide mechanistic evidence that VANGL2 modulates cellular mechanics, including FA complexes, actomyosin organization, actomyosin-mediated contractility and traction force generation via RhoA signaling (Figure 7). We identify a positive correlation between VANGL2 and YAP signaling and show that VANGL2 controls cell migration through its direct role in mechanosignaling. This work represents an important advance in our understanding of VANGL2 function by demonstrating an indispensable role for this core PCP component in mechanosignaling for the first time.

## Materials and Methods

### Animals

All animal maintenance and procedures were conducted in compliance with the requirements of the Animal (Scientific Procedures) Act 1986. Animal work was approved by the South Kensington and St Mary’s AWERB committee, Imperial College London. Mice were genotyped by MRC Harwell (Oxford, United Kingdom). *Vangl2*^*Lp/+*^ mice carry a heterozygous substitution mutation S464N that results in loss of function of VANGL2 (Kibar et al., 2001; Murdoch, 2001); WT littermates were used as controls. Mice were maintained on a C3H/HeH background (MRC Harwell, Oxford) and were housed in specific pathogen-free conditions and given food and water *ad libitum*. Male or female adult mice aged 8–12 weeks were used for alveolar and TEC isolation in this study; whereas PCLS were obtained from postnatal day 3 (P3) mouse lungs. Mice were humanely killed by intraperitoneal pentobarbital injection.

### Precision-Cut Lung Slicing and *in vitro* Culture

Precision-cut lung slices were obtained from WT and *Vangl2*^*Lp/+*^ littermate P3 mouse lungs as previously described (Akram et al., 2019a,b). Briefly, P3 mice were culled and the anterior chest and neck wall was excised. A small opening was made in the anterior wall of the trachea just below the cricoid cartilage. A 25G rigid metallic cannula was inserted through the trachea. The lungs were inflated by injecting 0.2 ml of 37°C warm, 1.5% low-gelling temperature agarose (Sigma–Aldrich) prepared with 1 × HBSS/HEPES buffer (Life Technologies). Agarose was injected to inflate both lungs keeping them *in situ* within the chest cavity at a volume that enabled lungs to be fully inflated without hyper- or sub-optimal inflation. After inflation, agarose was solidified by applying ice to the chest cavity for 1 min. The lungs were excised from the body along with heart and trachea and immersed in ice-cold serum-free (SF)-DMEM (Gibco), and kept on ice until slicing. Left lung lobes were isolated and cut transversely at 300 μm using an automated vibratome (Compresstome^®^ VF-300-0Z; Precisionary Instruments LLC) in ice-cold HBSS/HEPES buffer. Slices were obtained from the middle 2/3rd of the lobe, to ensure similar sized slices, and placed in a 24-well plate in ice-cold SF-DMEM for all experiments. PCLS were then incubated at 37°C for 2 h and washed twice with warm SF-DMEM to remove excess agarose from the tissue. Slices were incubated for a further 1 h in SF-DMEM at 37°C. These PCLS were then used for time-lapse imaging and *ex vivo* alveologenesis (Supplementary Figure S1A).

### Isolation of Mouse Alveolar Epithelial Cells

Mice were culled and metallic cannula was inserted through the trachea as described above. The lungs were gently flushed with PBS and 1 ml of Dispase (50 U/ml; Corning; Cat# 354235) was injected into the lungs, followed by 200 μl of 1% low-gelling temperature agarose (Sigma–Aldrich) dissolved in SF-DMEM (Gibco). After injection, ice was applied to the chest cavity for 1 min to solidify agarose and prevent Dispase leakage. Lungs were excised from the body. Tracheas were collected for isolation of trachea epithelial cells as described below. Heart and connective tissues were removed before immersing the lungs in 1.5 ml of ice-cold Dispase solution (50 U/ml; Corning), and incubated at room temperature for 45 min. Lung lobes were separated and connective tissues were discarded. Lung lobes were then homogenized using forceps in 3 ml of DMEM/HEPES/DNase medium [DMEM medium (Gibco) containing 25 mM HEPES (Gibco), 1% penicillin and streptomycin (Sigma–Aldrich), and 50 U/ml DNase I (Thermo Fisher Scientific)]. The homogenized mixture was sequentially passed through 100, 70, and 30 μm cell strainers into a 15 ml centrifuge tube. The cell suspension was centrifuged at 550 × *g* for 5 min at 4°C to pellet the cells. Supernatant and a translucent layer of residual agarose above the cell pellet were carefully removed. Cells were then resuspended in 1 ml of DMEM/HEPES/DNase medium to perform cell counting. Cells were centrifuged at 300 × *g* for 5 min at 4°C and resuspended in MACS buffer (Miltenyi Biotec) with appropriate amount of CD31 (Miltenyi Biotec; Cat# 130-097-418) and CD45 microbeads (Miltenyi Biotec; Cat# 130-052-301) and incubated at 4°C for 20 min. The cell suspension was then passed through the MACS magnetic column and unwanted endothelial (CD31) and hematopoietic cells (CD45) were magnetically labeled and removed from the cell suspension. An appropriate amount of CD326 (EpCAM) microbeads (Miltenyi Biotec; Cat# 130-105-958) was then added to the cell suspension and incubated at 4°C for 20 min. After incubation, EpCAM-positive AECs were eluted from the magnetic column. AEC suspension was centrifuged at 300 × *g* for 5 min at 4°C and was resuspended in an appropriate amount of AEC growth medium [DMEM/F-12 medium (Gibco) containing 15 mM HEPES (Gibco), 4 mM glutamine (Life Technologies), 0.03% NaHCO_3_ (Sigma–Aldrich), 0.25 μg/ml Fungizone (Gibco), 10 U penicillin-streptomycin (Sigma–Aldrich), 1 × Insulin-Transferrin-Selenium (ITS; Life Technologies), 0.1 μg/ml Cholera toxin (Sigma–Aldrich), 25 ng/ml epidermal growth factor (EGF; Sigma–Aldrich), 30 μg/ml bovine pituitary extract (BPE; Gibco), and 5% fetal bovine serum (FBS; Gibco)]. AECs were seeded into Lab-Tek Permanox chamber slides (VWR) pre-coated with 50 μg/ml rat-tail collagen I (Gibco; diluted with 0.02 M acetic acid) and grown at 37°C with 5% CO_2_. AECs were fixed for immunostaining after 72 h culture.

### Isolation of Mouse Tracheal Epithelial Cells

Mouse TECs were isolated as previously described (Lam et al., 2010). Tracheas were separated from the lung lobes into ice-cold SF-Ham’s F-12 medium (Gibco) and the esophagus and connective tissues were carefully removed using forceps. Tracheas were then cut lengthwise to expose the lumen. Three tracheas from mice of the same genotype were pooled for each sample. Tracheas were then incubated with SF-Ham’s F-12 medium (Gibco) containing 0.15% Pronase (Roche; Cat# 10165921001) in a 15 ml tube overnight at 4°C. Cells were gently dissociated from tracheas by inverting the tubes. Equal volume of Ham’s F-12 supplemented with 20% FBS was added and tubes were inverted several times. Tracheas were transferred to a new tube containing fresh Ham’s F-12 supplemented with 10% FBS and the tube was inverted several times. This step was repeated twice and then all supernatant was collected and centrifuged at 390 × *g* for 10 min at 4°C to pellet the cells before discarding the supernatant. Cells were gently resuspended in DNase I solution (0.5 mg/ml; Sigma–Aldrich; dissolved in SF-Ham’s F-12 medium) and incubated on ice for 5 min. Cells were then centrifuged at 390 × *g* for 5 min at 4°C with the slowest break speed and supernatant was discarded. Cells were then resuspended in TEC medium [DMEM/F-12 medium (Gibco) containing 15 mM HEPES, 4 mM glutamine, 0.03% NaHCO_3_, 0.25 μg/ml Fungizone, and 10 U penicillin-streptomycin] supplemented with 10% FBS, seeded onto a six-well plate and incubated at 37°C for 4 h. This allowed fibroblasts to attach to the dish while TECs remained floating in the supernatant. The supernatant was then collected, centrifuged at 390 × *g* for 5 min at 4°C, and cell pellets were resuspended in an appropriate amount of pre-warmed TEC Plus medium [TEC medium enriched with 1 × ITS, 0.1 μg/ml Cholera toxin, 25 ng/ml EGF, 30 μg/ml BPE, 5% FBS, and 0.1 μM retinoic acid (Sigma–Aldrich)] to perform cell counting. After cell counting, TECs were diluted with appropriate amount of TEC Plus medium and grown at 37°C with 5% CO_2_. All dishes and coverslips used to culture TECs were pre-coated with 50 μg/ml rat-tail collagen I (Gibco; diluted with 0.02 M acetic acid). TECs were fixed for immunostaining after 72 h culture.

### Culture of Human Alveolar Epithelial Cell Line A549

Human alveolar adenocarcinoma A594 cells were purchased from American Type Culture Collection (ATCC). Cells were grown at 37°C with 5% CO_2_ in DMEM (Gibco) containing 10% FBS.

### Live Staining of PCLS and Time-Lapse Imaging

P3 PCLS from WT and *Vangl2*^*Lp/+*^ mice were stained with FITC-conjugated EpCAM (EpCAM-FITC) antibody following the protocol described in Akram et al. (2019a). Briefly, PCLS were incubated for 1 h at 37°C in the dark with EpCAM-FITC antibody at 1:200 (Thermo Fisher Scientific; Cat# 11-5791-80) (500 μl antibody solution per well) prepared in phenol red-free SF-DMEM (Life Technologies). Slices were then washed three times with phenol red-free SF-DMEM. For time-lapse imaging, the EpCAM-FITC labeled PCLS were placed at the center of the well of an uncoated Ibidi 24-well μ-plate (Ibidi). Then, a 0.4 μm pore, 12 mm transwell (Corning) was placed onto the PCLS, with the rim of the transwell removed to allow the transwell filter to contact the slice at the bottom of the well. Image media, containing EpCAM-FITC at 1:500 in phenol red-free SF-DMEM, was then added to the upper chamber (500 μl) and bottom chamber (300 μl). A 1.66 g metal flat washer (M8-5/16th inches diameter) was placed on top of the transwell housing to keep the PCLS in place throughout the duration of imaging. The PCLS were then left in the incubator for 2 h, ensuring the cells were all labeled and allowing the PCLS to settle down prior to image acquisition. The 24-well plate was then transferred to a pre-equilibrated and humidified incubator chamber (37°C, 5% CO_2_ and air oxygen levels, ∼21%) of an inverted Zeiss Axio Observer widefield epifluorescence microscope. Imaging was conducted using a long working distance 40×/0.7NA air objective lens. In each experiment, a duplicate slice was imaged for each experimental group (WT and *Vangl2^*Lp/+*^)*. A maximum of four slices were imaged in a single time-lapse experiment. Images were captured using GFP filter, excitation 450–490 nm, emission 500–550 nm (for EpCAM-FITC) from four fields per slice for 13 h at 15 min intervals. Ten images were captured along the *z*-axis with 1 μm step-gap to make a z-stack from each slice. Channel color was changed during video post-processing to aid visualization.

### Cell Tracking Using Icy Software

Icy (version 1.9.8.0), an open source bioimaging analysis software, was used for cell tracking and migration quantification during time-lapse imaging following previously described protocol (Fabrice de Chaumont et al., 2012; Akram et al., 2019a). Briefly, raw time-lapse sequences from each field of a lung slice were acquired into Icy and EpCAM-positive cells were tracked. Results were manually verified to ensure tracking was correct. EpCAM-FITC-positive cells were tracked for quantification of epithelial cell migration. FITC-channel was selected from best-focused slice from a z-stack for tracking. Cell migration in the *X*–*Y* plane only (not the *Z*-plane) was recorded. Two aspects of tracking data were considered: (1) Net cell migration during time-lapse period (13 h), in which the mean value was calculated to present how much linear distance in the *X*–*Y* axis (“Start” point to “End” point) a cell had migrated (Akram et al., 2019b). A mean value of migrated distances of total number of cells from each field is presented as “mean net cell migration.” (2) The net distance migrated by individual cells in a field within the specified time period in each experimental group was also investigated. To determine the proportion of highly motile cells, the percentage of cells that migrated between 5 and 11 μm per field within a defined time period (13 h) was quantified. Two to four fields from each slice were analyzed. Three independent experiments were conducted using three mice per group.

### PCLS *in vitro* Culture

P3 PCLS from WT and *Vangl2*^*Lp/+*^ mice were cultured in SF-DMEM for 72 h (Supplementary Figure S1A) following a previously described protocol (Akram et al., 2019a). Briefly, unlabeled 300 μm thick PCLS from P3 WT and *Vangl2*^*Lp/+*^ mice were cultured in SF-DMEM in a 24-well plate (1 PCLS per well in 1 ml medium) for 72 h under normal culture conditions (37°C, 5% CO_2_). Medium was changed every alternate day. Slices were fixed with 10% formalin for 30 min at room temperature for histology sectioning and morphometric analysis.

### Morphometric Analysis of PCLS

To evaluate alveologenesis, mean linear intercept (Lm) and the number of airspaces were quantified in H&E-stained P3 PCLS cultured in SF-DMEM for 72 h (Poobalasingam et al., 2017). P3 WT and *Vangl2*^*Lp/+*^ PCLS from 0 and 72 h culture were fixed as above, embedded in paraffin, keeping the slice surfaces horizontal to the block and sectioned at 4 μm thickness. Sections were deparaffinized and stained with H&E. Images were captured using a Leica DM2500 widefield microscope and a ×20, 0.7 NA objective lens. For quantification of Lm, a grid of eight horizontal lines was superimposed on images from H&E sections, using FIJI (Schindelin et al., 2012; Poobalasingam et al., 2017). The number of times alveolar cells/walls intercepted the line was counted and Lm was calculated using the following equation: Lm = *NL*/*X*, where *N* = number of lines counted, *L* = length of line, and *X* = total number of intercepts counted. Three fields per lung section, and three sections per PCLS from three mice for each group were imaged. The number of alveolar spaces/airspace per field was counted and presented as number of airspaces per millimeter square area. Alveolar spaces were quantified from the same visual fields used for Lm quantification. Fields of view containing blood vessels or airways were omitted from analysis.

### MTT Assay

To assess viability of the cells within PCLS, for each experiment, similar size PCLS were placed into wells of a 24-well plate in duplicate (1 slice per well). MTT assay (Sigma–Aldrich) was performed according to manufacturer’s instructions and protocol described in Akram et al. (2019a). Briefly, 500 μl of 10% MTT solution prepared in SF-DMEM was added per well, per slice and incubated at 37°C for 1 h. Formazan crystals that formed within the viable cells were solubilized by adding an equal volume of DMSO for 10 min at 37°C. 200 μl of eluted formazan solution from each slice was placed into individual wells of a 96-well plate. Absorbance (OD) was measured at 570 nm and corrected at 690 nm using a plate reader. MTT was performed on post time-lapse PCLS and on 72 h *ex vivo* cultured PCLS.

### siRNA Transfection

siRNA transfection was performed on A549 cells using DharmaFECT 1 Transfection Reagent (Dharmacon) according to the manufacturer’s instructions. A549 cells were cultured in DMEM (Gibco) containing 10% FBS for 18 h before siRNA transfection. Cells were transfected with a final concentration of 20 nM siRNA and cultured for further 48 h before immunostaining or RNA extraction for qRT-PCR analysis. Four independent experiments were conducted. Non-targeting (control) and *VANGL2* siRNA oligos used in this study are as follows: ON-TARGETplus VANGL2 siRNA (Dharmacon; Cat# L-010581-00-0020) and siGENOME non*-*targeting siRNA pool #2 (Dharmacon; Cat# D-001206-14-20).

### Scratch Wound Assay

A total of 50,000 WT and *Vangl2*^*Lp/+*^ TECs in TEC Plus medium (described above) were seeded on rat-tail collagen I (Gibco)-coated coverslips (diameter: 13 mm; Sarstedt) and medium was changed after 48 h. After culturing for 72 h, a single scratch wound was made across the center of confluent TEC monolayers using a 200 μl pipette tip. Wells were gently washed with PBS to remove floating cells and replaced with fresh TEC Plus medium. Wells were imaged at *t* = 0 using an inverted Zeiss Axio Observer widefield epifluorescence microscope in a pre-equilibrated and humidified incubator chamber (37°C, 5% CO_2_ and air oxygen levels, ∼21%). Images were taken using an EC Plan-Neofluar 10x/0.30 objective lens and tiling method was used to image the entire coverslip. Cells were then cultured for a further 20 h and images were taken using the same protocol at *t* = 20 h. For WNT5A treatment, *Vangl2*^*Lp/+*^ TECs were cultured with TEC Plus medium supplemented with 1 μg/ml recombinant WNT5A (R&D Systems; Cat# 645-WN). The percentage of wound healed was calculated by drawing around the outline of the scratch using the freehand tool in FIJI and subtracting the area at *t* = 20 h from the area at *t* = 0 h. Four independent experiments were conducted, and each individual experiment contained pooled TECs from three mice per sample.

For actomyosin inhibitor studies, A549 cells were seeded at 40,000 cells/well in eight-well chamber slides (VWR) pre-coated with 50 μg/ml rat-tail collagen I (Gibco) and cultured for 48 h in DMEM supplemented with 10% FBS until cells reached confluence. Cells were then serum starved for 24 h before treatment with 25 μM blebbistastin (Cambridge Bioscience; Cat# CAY13891) or 0.5 μM cytochalasin D (Sigma–Aldrich; Cat# C8273) diluted in DMEM supplemented with 0.5% FBS. Since blebbistatin and cytochalasin D were reconstituted using DMSO and the final concentrations of DMSO in the working media were 0.08 and 0.025%, respectively. Control media for blebbistatin and cytochalasin D in scratch assays was 0.5% FBS-DMEM containing 0.08 and 0.025% DMSO, respectively. Scratch assays, wound imaging, and measurement of wound healed were performed as detailed above.

### Quantification of Polarized Cells

Tracheal epithelial cells were fixed 20 h post-scratch and dual stained with a Golgi marker, GM130 (1:200; Becton Dickinson Biosciences; Cat# 610822) and DAPI (1:500; Sigma; Cat# D9542) that labeled the nuclei. Detailed immunocytochemistry protocol is described below. After staining, images were captured on a Leica SP8 inverted confocal microscope using a HC PL APO 10x/0.40 objective lens. Cell directionality during migration was quantified as previously described (Etienne-Manneville and Hall, 2001; Cory, 2011; Poobalasingam et al., 2017). Using the line tool in FIJI, a cross with a 120° arc facing the direction of migration was drawn from the center of the nucleus in cells at the leading edge. Golgi were considered polarized if they were located within the 120° arc facing the direction of migration; whereas cells with the entire or majority of the Golgi localized outside the 120° arc were classed as non-polarized. Golgi orientation was quantified from four independent experiments, with each individual experiment containing pooled TECs from three mice per sample.

### Immunohistochemistry

Lung tissues were fixed in 4% paraformaldehyde (PFA), dehydrated in ethanol and embedded in paraffin, and sectioned at 4 μm thickness. Sections were deparaffinized and rehydrated using standard protocols: Gentaclear and descending ethanol concentrations. Sections were then incubated in 3% H_2_O_2_ for 5 min at room temperature, followed by 1 h blocking with PBS containing horse serum provided by the Vectastain Elite ABC kit (Vector Labs) at room temperature, and incubation with rabbit anti-YAP antibody (1:400; Cell Signaling Technology; Cat# 14074) overnight at 4°C. Next, sections were incubated with biotinylated antibody from the Vectastain Elite ABC HRP kit for 30 min at RT, followed by Vectastain Elite ABC reagent mixture for 30 min at RT. Then, sections were incubated with peroxidase substrate, diaminobenzidine (DAB) (BD Bioscience) following the manufacturers’ instructions. Stain development was quenched by rinsing slides in tap water. Slides were counterstained with hematoxylin and mounted with DPX mountant (Solmedia). Images were taken on a Leica DM2500 widefield microscope with a 40× objective lens. The total number of cells and the number of nuclear YAP-positive cells in the airways were quantified using FIJI software. Three mice were analyzed per genotype and eight airways were quantified per mouse. Negative controls, where the primary antibody was omitted, were included in each experiment.

### Immunofluorescence

Cells were seeded on rat-tail collagen I-coated Lab-Tek Permanox chamber slide (VWR) or coverslips (diameter: 13 mm; Sarstedt). For Rho activation experiment, TECs were treated with 1 μg/ml of Rho activator II, CN03 (Cytoskeleton Inc; Cat# CN03–A) for 3 h. For treatment with actomyosin inhibitors, A549 cells were treated with blebbistastin (Cambridge Bioscience; Cat# CAY13891) at 25 μM for 30 min, ROCK inhibitor Y-27632 (Sigma–Aldrich; Cat# Y0503) at 10 μM for 1 h, or cytochalasin D (Sigma–Aldrich; Cat# C8273) at 0.5 μM for 30 min. Since blebbistatin and cytochalasin D were reconstituted using DMSO, media containing DMSO were used as control. Cells were fixed in 4% PFA for 10 min at RT, washed in PBS, permeabilized with 0.2% Triton X-100 in PBS at RT for 5 min, followed by 1 h blocking with PBSBT (3% BSA, 0.1% Triton X-100 in PBS) at RT for 1 h. After blocking, cells were incubated with primary antibodies diluted in PBSBT blocking buffer at 4°C overnight. Primary antibodies used in this study are as follows: rabbit anti-paxillin Y113 (1:100; Abcam; Cat# ab32084), rabbit anti-pFAK Tyr397 (1:100; CST; Cat# 3283), rabbit anti-pMLC2 Ser19 (1:50; CST; Cat# 3671), rabbit anti-GM130 (1:200; BD Biosciences; Cat# 610822), mouse anti-pan-cytokeratin (1:200; Sigma–Aldrich; C2931). After five washes in PBS, cells were then incubated with rhodamine phalloidin (1:100; Biotium; Cat# 00027) and species-specific Alexa Fluor 488 and 647 secondary antibodies (1:500; Thermo Fisher Scientific) at RT for 1 h. After washing in PBS, cell nuclei were labeled with DAPI (Sigma–Aldrich). Coverslips were then mounted with ProLong Gold Antifade Mountant (Invitrogen; Cat# P36930). Cells were imaged on a Leica SP8 inverted confocal microscope using a HC PL APO 40x/1.30 oil objective lens. For quantification of polarized cells experiment, images were captured with a HC PL APO 10x/0.40 air objective lens.

Lung cryosections from E18.5 WT, *Vangl2*^*Lp/+*^, and *Vangl2*^*Lp/Lp*^ mice were immunostained with mouse anti-pan-cytokeratin (1:200; Sigma–Aldrich; C2931) and rabbit anti-pYAP Ser127 (1:100; CST; Cat# 4911). Sections were then incubated with species-specific Alexa Fluor 488 and 647 secondary antibodies (1:500; Thermo Fisher Scientific). Cell nuclei were labeled with DAPI (Sigma–Aldrich) and sections were then mounted with ProLong Gold Antifade Mountant (Invitrogen; Cat# P36930). Lung airways were imaged on a Leica SP8 inverted confocal microscope using a HC PL APO 40x/1.30 oil objective lens. For quantification of cytoplasmic pYAP levels, images of the nuclei stained with DAPI were thresholded, resulting in a binary mask of the nuclei. This mask was overlaid on the pYAP immunofluorescence image and subtracted from the pYAP mask. The mean fluorescent intensity was measured giving the average YAP expression in the cytoplasm.

For immunofluorescent staining of fixed PCLS, first P3 PCLS were dual stained as above with EpCAM-FITC (1:200; Thermo Fisher Scientific; Cat# 11-5791-80) and silicon rhodamine far-red fluorophore-conjugated DNA minor groove binder bisbenzimide (SiR-DNA) at (1:300; Tebu-Bio Ltd.; Cat# SC007). PCLS were fixed with 10% buffered formalin then blocked and permeabilized with PBSBT blocking buffer (1% BSA, 0.5% Triton X-100 in PBS) in the dark for 1 h at RT. Samples were then incubated with anti-mouse CD11c-PE conjugated primary antibody (1:200; Biolegend; Cat# 117307) overnight at 4°C to label macrophages. After incubation, EpCAM-FITC, CD11c-PE, and SiR-DNA triple-labeled PCLS were washed and mounted onto glass slides with ProLong Gold Antifade Mountant (Invitrogen; Cat# P36930). Images were taken using a Zeiss Axio Observer inverted microscope, with Lumencor Spectra X LED light source and Hamamatsu Flash 4.0 camera, using 40×/0.7 NA, air objective lens. The z-stacks of images were deconvolved as above and best-focused single slice images were analyzed. For some experiments, channel colors were changed during image post-processing to aid visualization.

### Quantification of Focal Adhesions

Cell FAs were imaged on a Leica SP8 inverted confocal microscope using a HC PL APO 40x/1.30 oil objective lens. Quantification of FAs was performed using FIJI software. First, a raw image was split into three channels: DAPI, Alexa Fluor 488-conjugated paxillin, and rhodamine-conjugated phalloidin. The single channel paxillin image was thresholded to highlight the paxillin staining and “analyze particles” tool was used to measure the size of paxillin (FAs) in the unit of micron^2^. A size filter of “> 0.1 micron^2^ to infinity” was used to exclude non-specific noise. A list of measurements was produced and each of them represented a single FA size. The result was exported to Excel and the mean FA size was calculated using the Excel “average” function. The total FA area was also calculated by summing all the FA areas. For the quantification of FA density, phalloidin channel was also analyzed to measure the total cell area (micron^2^). “Threshold” tool was used to exclude the cell-free area. Any noise outside the cells was manually removed using the drawing tool. Cell areas were then selected and “measure” tool in FIJI was used, which produced the total cell area in micron^2^. The result was exported to the Excel. The FA density = total FA area normalized to total cell area.

### RNA Extraction and Quantitative RT-PCR

Total RNA was extracted from WT, *Vangl2*^*Lp/+*^, and *Vangl2*^*Lp/Lp*^ mouse lung tissues and A549 cells using the RNeasy mini kit (Qiagen) according to the manufacturer’s protocols. For mouse lung tissues, a two-step homogenization was carried out using a FastPrep-24^TM^ Tissue Homogenizer (MP Biomedicals) followed by a QIAshredder kit (Qiagen) following the manufacturer’s instructions. RNA concentration and quality was assessed using the TapeStation 2200 (Agilent). 1 μg of total RNA was reverse-transcribed to cDNA using the High-Capacity cDNA Reverse Transcription kit (Applied Biosystems). Quantitative RT-PCR (qRT-PCR) was performed using TaqMan Fast Advanced Master Mix (Life Technologies) and run on a Viia7 Real-Time PCR System (Applied Biosystems). For mouse cDNA, *B2m* and *Hprt* were used as reference genes; whereas *B2M* and *GUSB* were used as reference genes for human cDNA. Relative transcript levels were analyzed using the 2^–ΔΔ*CT*^ method. Three mice were used per group and all samples were tested in triplicate. All primers used in this study were purchased from Life Technologies as follows: *Ctgf* (Mm01192933_g1), *Cyr61* (Mm00487498_m1), *Ankrd1* (Mm00496512_m1), *B2m* (Mm00437762_m1), *Hprt* (Mm03024075_m1), *B2M* (Hs00187842_m1), *GUSB* (Hs00939627_m1), and *VANGL2* (Hs00393412_m1).

### Protein Extraction and Western Blotting

A549 cells were lyzed in lysis buffer containing 0.1% Triton X-100 (Sigma–Aldrich), 1 × protease inhibitor cocktail (PIC; Roche), 1 × PhosSTOP^TM^ (Roche), 1 mM phenylmethylsulfonyl fluoride (PMSF; Sigma–Aldrich), and 5 μg/ml sodium deoxycholate (Sigma–Aldrich) in PBS for 15 min. Lysates were collected with a cell scraper and sonicated for 5 s. Lysates were then centrifuged at 4°C, 14,000 × *g* for 30 min and supernatant was collected and stored at −80°C until use. The concentrations of protein samples were quantified using the Pierce BCA Protein Assay Kit (Thermo Fisher Scientific) according to the manufacturer’s instructions. Protein samples were subjected to SDS-PAGE and transferred onto polyvinylidene difluoride (PVDF) membranes (Thermo Fisher Scientific). After blocking with TBSBT blocking buffer (0.1% Tween-20, 5% BSA in TBS) for 1 h at RT, membranes were incubated with rabbit anti-phospho-FAK Y397 (1:1000; CST; Cat# 3283) and mouse anti-FAK (1:3000; CST; Cat# 05-537) primary antibodies overnight at 4°C. After washing in TBS containing 0.1% Tween-20, membranes were incubated with IRDye 800CW goat anti-rabbit IgG secondary antibody (1:15,000; LI-COR Biosciences; Cat# 925-32211) and IRDye 680RD goat anti-mouse IgG secondary antibody (1:10,000; LI-COR Biosciences; Cat# 925-68070) at RT for 1 h. Immunodetection was performed using an Odyssey infrared imaging system (LI-COR) and western blots were analyzed using the LI-COR Image Studio Lite Software.

### Elastic Pillar Arrays

Elastic micropillar arrays were fabricated in PDMS as previously described (Chronopoulos et al., 2016). Briefly, PDMS (Sylgard 184, Dow Corning) was mixed with its curing agent in a 10:1 ratio according to the manufacturer’s specification, poured on the silicon mold, and cured at 60°C for 1 h. After curing, pillars were peeled from the mold in PBS and store at 4°C. Prior to cell seeding, pillars were coated with 50 μg/ml rat-tail collagen I diluted in 0.02 M acetic acid and incubated at 37°C for 1 h. After incubation, collagen solution covering the pillars was carefully replaced with PBS followed by TEC Plus medium. TECs were then counted and seeded on the pillar microarrays submerged in TEC Plus medium and incubated at 37°C at 5% CO_2_ for 24 h before analysis. Time-lapse imaging (1 frame per second for 1 min) of individual cells was acquired on an inverted microscope (Eclipse Ti; Nikon) with a sCMOS camera (Neo sCMOS Andor) in a pre-equilibrated and humidified incubator chamber. Each sample was analyzed for a maximum of 30 min to ensure cell viability. Data were analyzed with a custom MATLAB script to track individual pillar deflection, and a cell-free area was used to subtract stage drift. Traction forces were calculated from pillar deflections using the spring constant (*k* = 1.36 nN/μm) of the pillars.

### Quantification and Statistical Analysis

All graphs and statistical tests were produced in GraphPad Prism 8. All data are presented as mean ± standard error of mean (SEM). D’Agostino–Pearson test was performed to determine the normality of data. Non-parametric datasets were analyzed using Mann–Whitney *U*-test (comparison of two groups), or Kruskal–Wallis with Dunn’s multiple comparisons test (comparison of more than two groups). Normally distributed datasets were analyzed using two-tailed unpaired Student’s *t*-test (comparison of two groups), or one-way ANOVA with Tukey’s *post hoc* test (comparison of more than two groups). Details of the statistical tests used, the value of *n*, and number of experiments performed are all detailed in the figure legends. *P* < 0.05 was considered statistically significant.

## Data Availability Statement

The raw data supporting the conclusions of this article will be made available by the authors, without undue reservation.

## Ethics Statement

The animal study was reviewed and approved by the South Kensington and St Mary’s AWERB Committee, Imperial College London.

## Author Contributions

S-SC, CD, and MG designed the study and supervised the project. S-SC, KA, CM, and SK performed experiments. S-SC, KA, and CM analyzed the data. S-SC, KA, CM, MG, and CD wrote the manuscript. DG processed the movies and provided imaging expertise. CD, MG, AR, and MH provided conceptual advice. MH and CD acquired funding. All authors discussed the results and contributed to editing the manuscript.

## Conflict of Interest

The authors declare that the research was conducted in the absence of any commercial or financial relationships that could be construed as a potential conflict of interest.

## References

[B1] Abreu-Blanco M. T. Watts J. J. Verboon J. M. Parkhurst S. M. (2012). Cytoskeleton responses in wound repair. *Cell. Mol. Life Sci.* 69 2469–2483. 10.1007/s00018-012-0928-2 22349211 PMC3388155

[B2] Akram K. M. Yates L. L. Mongey R. Rothery S. Gaboriau D. C. A. Sanderson J. (2019a). Live imaging of alveologenesis in precision-cut lung slices reveals dynamic epithelial cell behaviour. *Nat. Commun.* 10:1178. 10.1038/s41467-019-09067-3 PMC6414680 30862802

[B3] Akram K. M. Yates L. L. Mongey R. Rothery S. Gaboriau D. C. Sanderson J. (2019b). Time-lapse imaging of alveologenesis in mouse precision-cut lung slices. *Bio Protoc.* 9:e3403. 10.21769/BioProtoc.3403 PMC7853931 33654904

[B4] Amano M. Ito M. Kimura K. Fukata Y. Chihara K. Nakano T. (1996). Phosphorylation and activation of myosin by Rho-associated kinase (Rho- kinase). *J. Biol. Chem.* 271 20246–20249. 10.1074/jbc.271.34.20246 8702756

[B5] Andre P. Wang Q. Wang N. Gao B. Schilit A. Halford M. M. (2012). The Wnt coreceptor Ryk regulates Wnt/planar cell polarity by modulating the degradation of the core planar cell polarity component Vangl2. *J. Biol. Chem.* 287 44518–44525. 10.1074/jbc.M112.414441 23144463 PMC3531765

[B6] Aw W. Heck B. Joyce B. Devenport D. (2016). Transient tissue-scale deformation coordinates alignment of planar cell polarity junctions in the mammalian skin. *Curr. Biol.* 26 2090–2100. 10.1016/j.cub.2016.06.030 27451904 PMC5005808

[B7] Baarsma H. A. Skronska-Wasek W. Mutze K. Ciolek F. Wagner D. E. John-Schuster G. (2017). Noncanonical WNT-5A signaling impairs endogenous lung repair in COPD. *J. Exp. Med.* 214 143–163. 10.1084/jem.20160675 27979969 PMC5206496

[B8] Babayeva S. Zilber Y. Torban E. (2011). Planar cell polarity pathway regulates actin rearrangement, cell shape, motility, and nephrin distribution in podocytes. *Am. J. Physiol. Renal Physiol.* 300 F549–F560. 10.1152/ajprenal.00566.2009 20534871

[B9] Balaban N. Q. Schwarz U. S. Riveline D. Goichberg P. Tzur G. Sabanay I. (2001). Force and focal adhesion assembly: a close relationship studied using elastic micropatterned substrates. *Nat. Cell Biol.* 3 466–472. 10.1038/35074532 11331874

[B10] Belotti E. Puvirajesinghe T. M. Audebert S. Baudelet E. Camoin L. Pierres M. (2012). Molecular characterisation of endogenous Vangl2/Vangl1 heteromeric protein complexes. *PLoS One* 7:e46213. 10.1371/journal.pone.0046213 23029439 PMC3460870

[B11] Beningo K. A. Dembo M. Kaverina I. Small J. V. Wang Y. L. (2001). Nascent focal adhesions are responsible for the generation of strong propulsive forces in migrating fibroblasts. *J. Cell Biol.* 153 881–887. 10.1083/jcb.153.4.881 11352946 PMC2192381

[B12] Choi H. J. Zhang H. Park H. Choi K. S. Lee H. W. Agrawal V. (2015). Yes-associated protein regulates endothelial cell contact-mediated expression of angiopoietin-2. *Nat. Commun.* 6:6943. 10.1038/ncomms7943 25962877

[B13] Chronopoulos A. Robinson B. Sarper M. Cortes E. Auernheimer V. Lachowski D. (2016). ATRA mechanically reprograms pancreatic stellate cells to suppress matrix remodelling and inhibit cancer cell invasion. *Nat. Commun.* 7:12630. 10.1038/ncomms12630 27600527 PMC5023948

[B14] Cory G. (2011). Scratch-wound assay. *Methods Mol. Biol.* 769 25–30. 10.1007/978-1-61779-207-6_2 21748666

[B15] Davey C. F. Moens C. B. (2017). Planar cell polarity in moving cells: think globally, act locally. *Development* 144 187–200. 10.1242/dev.122804 28096212 PMC5394761

[B16] de Chaumont F. Dallongeville S. Chenouard N. Hervé N. Pop S. Provoost T. (2012). Icy: an open bioimage informatics platform for extended reproducible research. *Nat. Methods* 9 690–696. 10.1038/nmeth.2075 22743774

[B17] Dobrokhotov O. Samsonov M. Sokabe M. Hirata H. (2018). Mechanoregulation and pathology of YAP/TAZ via Hippo and non-Hippo mechanisms. *Clin. Transl. Med.* 7:23. 10.1186/s40169-018-0202-9 PMC6087706 30101371

[B18] Duan X. Gao Y. Liu Y. (2017). Ryk regulates Wnt5a repulsion of mouse corticospinal tract through modulating planar cell polarity signaling. *Cell Discov.* 3:17015. 10.1038/celldisc.2017.15 28660073 PMC5475318

[B19] Dupont S. Morsut L. Aragona M. Enzo E. Giulitti S. Cordenonsi M. (2011). Role of YAP/TAZ in mechanotransduction. *Nature* 474 179–184. 10.1038/nature10137 21654799

[B20] Enomoto M. Hayakawa S. Itsukushima S. Ren D. Y. Matsuo M. Tamada K. (2009). Autonomous regulation of osteosarcoma cell invasiveness by Wnt5a/Ror2 signaling. *Oncogene* 28 3197–3208. 10.1038/onc.2009.175 19561643

[B21] Etienne-Manneville S. Hall A. (2001). Integrin-mediated activation of Cdc42 controls cell polarity in migrating astrocytes through PKCζ. *Cell* 106 489–498. 10.1016/s0092-8674(01)00471-8 11525734

[B22] Evans E. A. Calderwood D. A. (2007). Forces and bond dynamics in cell adhesion. *Science* 316 1148–1154. 10.1126/science.1137592 17525329

[B23] Ezzie M. E. Crawford M. Cho J.-H. Orellana R. Zhang S. Gelinas R. (2012). Gene expression networks in COPD: microRNA and mRNA regulation. *Thorax* 67 122–131. 10.1136/thoraxjnl-2011-200089 21940491

[B24] Friedl P. Gilmour D. (2009). Collective cell migration in morphogenesis, regeneration and cancer. *Nat. Rev. Mol. Cell Biol.* 10 445–457. 10.1038/nrm2720 19546857

[B25] Gao B. Song H. Bishop K. Elliot G. Garrett L. English M. A. (2011). Wnt signaling gradients establish planar cell polarity by inducing Vangl2 phosphorylation through Ror2. *Dev. Cell* 20 163–176. 10.1016/j.devcel.2011.01.001 21316585 PMC3062198

[B26] Geiger B. Spatz J. P. Bershadsky A. D. (2009). Environmental sensing through focal adhesions. *Nat. Rev. Mol. Cell Biol.* 10 21–33. 10.1038/nrm2593 19197329

[B27] Goffin J. M. Pittet P. Csucs G. Lussi J. W. Meister J. J. Hinz B. (2006). Focal adhesion size controls tension-dependent recruitment of α-smooth muscle actin to stress fibers. *J. Cell Biol.* 172 259–268. 10.1083/jcb.200506179 16401722 PMC2063555

[B28] Goodrich L. V. Strutt D. (2011). Principles of planar polarity in animal development. *Development* 138 1877–1892. 10.1242/dev.054080 21521735 PMC3082295

[B29] Hamadi A. Bouali M. Dontenwill M. Stoeckel H. Takeda K. Rondé P. (2005). Regulation of focal adhesion dynamics and disassembly by phosphorylation of FAK at tyrosine 397. *J. Cell Sci.* 118 4415–4425. 10.1242/jcs.02565 16159962

[B30] Henderson D. J. Long D. A. Dean C. H. (2018). Planar cell polarity in organ formation. *Curr. Opin. Cell Biol.* 55 96–103. 10.1016/j.ceb.2018.06.011 30015152

[B31] Hopkins A. M. Pineda A. D. A. Winfree L. M. Brown G. T. Laukoetter M. G. Nusrat A. (2019). Organized migration of epithelial cells requires control of adhesion and protrusion through Rho kinase effectors. *Am. J. Physiol. Gastrointest. Liver Physiol.* 292 G806–G817. 10.1152/ajpgi.00333.2006 17138966

[B32] Isago H. Mitani A. Mikami Y. Horie M. Urushiyama H. Hamamoto R. (2020). Epithelial expression of YAP and TAZ is sequentially required in lung development. *Am. J. Respir. Cell Mol. Biol.* 62 256–266. 10.1165/rcmb.2019-0218oc 31486675

[B33] Jaalouk D. E. Lammerding J. (2009). Mechanotransduction gone awry. *Nat. Rev. Mol. Cell Biol.* 10 63–73. 10.1038/nrm2597 19197333 PMC2668954

[B34] Képiró M. Várkuti B. H. Végner L. Vörös G. Hegyi G. Varga M. (2014). Para-nitroblebbistatin, the non-cytotoxic and photostable myosin II inhibitor. *Angew. Chem. Int. Ed.* 53 8211–8215. 10.1002/anie.201403540 24954740

[B35] Kibar Z. Vogan K. J. Groulx N. Justice M. J. Underhill D. A. Gros P. (2001). *Ltap*, a mammalian homolog of *Drosophila Strabismus/Van Gogh*, is altered in the mouse neural tube mutant Loop-tail. *Nat. Genet.* 28 251–255. 10.1038/90081 11431695

[B36] Kim H. Y. Nelson C. M. (2012). Extracellular matrix and cytoskeletal dynamics during branching morphogenesis. *Organogenesis* 8 56–64. 10.4161/org.19813 22609561 PMC3429513

[B37] Komiya Y. Habas R. (2008). Wnt signal transduction pathways. *Organogenesis* 4 68–75. 10.4161/org.4.2.5851 19279717 PMC2634250

[B38] Lacanna R. Wolfson M. R. Tian Y. Lacanna R. Liccardo D. Zhang P. (2019). Yap/Taz regulate alveolar regeneration and resolution of lung inflammation. *J. Clin. Invest.* 129 2107–2122. 10.1172/jci125014 30985294 PMC6486331

[B39] Lam H. C. Choi A. M. K. Ryter S. W. (2010). Isolation of mouse respiratory epithelial cells and exposure to experimental cigarette smoke at air liquid interface. *J. Vis. Exp.* 48:2513. 10.3791/2513 21372793 PMC3197407

[B40] Li C. Smith S. M. Peinado N. Gao F. Li W. Lee M. K. (2020). WNT5a-ROR signaling is essential for alveologenesis. *Cells* 9:384. 10.3390/cells9020384 32046118 PMC7072327

[B41] Lin C. Yao E. Zhang K. Jiang X. Croll S. Thompson-Peer K. (2017). YAP is essential for mechanical force production and epithelial cell proliferation during lung branching morphogenesis. *eLife* 6:e21130. 10.7554/eLife.21130 28323616 PMC5360446

[B42] Mayor R. Carmona-Fontaine C. (2010). Keeping in touch with contact inhibition of locomotion. *Trends Cell Biol.* 20 319–328. 10.1016/j.tcb.2010.03.005 20399659 PMC2927909

[B43] Mitra S. K. Hanson D. A. Schlaepfer D. D. (2005). Focal adhesion kinase: in command and control of cell motility. *Nat. Rev. Mol. Cell Biol.* 6 56–68. 10.1038/nrm1549 15688067

[B44] Montcouquiol M. Sans N. Huss D. Kach J. Dickman J. D. Forge A. (2006). Asymmetric localization of Vangl2 and Fz3 indicate novel mechanisms for planar cell polarity in mammals. *J. Neurosci.* 26, 5265–5275. 10.1523/JNEUROSCI.4680-05.2006 16687519 PMC6674235

[B45] Munoz-Soriano V. Belacortu Y. Paricio N. (2012). Planar cell polarity signaling in collective cell movements during morphogenesis and disease. *Curr. Genomics* 13 609–622. 10.2174/138920212803759721 23730201 PMC3492801

[B46] Murdoch J. N. (2001). Severe neural tube defects in the loop-tail mouse result from mutation of Lpp1, a novel gene involved in floor plate specification. *Hum. Mol. Genet.* 10 2593–2601. 10.1093/hmg/10.22.2593 11709546

[B47] Nabhan A. N. Brownfield D. G. Harbury P. B. Krasnow M. A. Desai T. J. (2018). Single-cell Wnt signaling niches maintain stemness of alveolar type 2 cells. *Science* 359 1118–1123. 10.1126/science.aam6603 29420258 PMC5997265

[B48] Nishita M. Enomoto M. Yamagata K. Minami Y. (2010). Cell/tissue-tropic functions of Wnt5a signaling in normal and cancer cells. *Trends Cell Biol.* 20 346–354. 10.1016/j.tcb.2010.03.001 20359892

[B49] Oakes P. W. Gardel M. L. (2014). Stressing the limits of focal adhesion mechanosensitivity. *Curr. Opin. Cell Biol.* 30 68–73. 10.1016/j.ceb.2014.06.003 24998185 PMC4459577

[B50] Pandya P. Orgaz J. L. Sanz-Moreno V. (2017). Actomyosin contractility and collective migration: may the force be with you. *Curr. Opin. Cell Biol.* 48 87–96. 10.1016/j.ceb.2017.06.006 28715714 PMC6137077

[B51] Park H. W. Kim Y. C. Yu B. Moroishi T. Mo J. S. Plouffe S. W. (2015). Alternative Wnt signaling activates YAP/TAZ. *Cell* 162 780–794. 10.1016/j.cell.2015.07.013 26276632 PMC4538707

[B52] Pieretti A. C. Ahmed A. M. Roberts J. D. Kelleher C. M. (2014). A novel in vitro model to study alveologenesis. *Am. J. Respir. Cell Mol. Biol.* 50 459–469. 10.1165/rcmb.2013-0056OC 24066869 PMC3930945

[B53] Poobalasingam T. Yates L. L. Walker S. A. Pereira M. Gross N. Y. Ali A. (2017). Heterozygous Vangl2 Looptail mice reveal novel roles for the planar cell polarity pathway in adult lung homeostasis and repair. *Dis. Models Mech.* 10 409–423. 10.1242/dmm.028175 28237967 PMC5399569

[B54] Pryor S. E. Massa V. Savery D. Andre P. Yang Y. Greene N. D. (2014). Vangl-dependent planar cell polarity signalling is not required for neural crest migration in mammals. *Development* 141, 3153–3158. 10.1242/dev.111427 25038043 PMC4197537

[B55] Qian D. Jones C. Rzadzinska A. Mark S. Zhang X. Steel K. P. (2007). Wnt5a functions in planar cell polarity regulation in mice. *Dev. Biol.* 306 121–133. 10.1016/j.ydbio.2007.03.011 17433286 PMC1978180

[B56] Raman R. Pinto C. S. Sonawane M. (2018). Polarized organization of the cytoskeleton: regulation by cell polarity proteins. *J. Mol. Biol.* 430 3565–3584. 10.1016/j.jmb.2018.06.028 29949753

[B57] Rao-Bhatia A. Zhu M. Yin W.-C. Coquenlorge S. Zhang X. Woo J. (2020). Hedgehog-activated Fat4 and PCP pathways mediate mesenchymal cell clustering and villus formation in gut development. *Dev. Cell* 52 647–658. 10.1016/j.devcel.2020.02.003 32155439

[B58] Schiller H. B. Fässler R. (2013). Mechanosensitivity and compositional dynamics of cell-matrix adhesions. *EMBO Rep.* 14 509–519. 10.1038/embor.2013.49 23681438 PMC3674437

[B59] Schindelin J. Arganda-Carreras I. Frise E. Kaynig V. Longair M. Pietzsch T. (2012). Fiji: an open-source platform for biological-image analysis. *Nat. Methods* 9 676–682. 10.1038/nmeth.2019 22743772 PMC3855844

[B60] Schliwa M. (1982). Action of cytochalasin D on cytoskeletal networks. *J. Cell Biol*. 92 79–91. 10.1083/jcb.92.1.79 7199055 PMC2112008

[B61] Schwarz U. S. Balaban N. Q. Riveline D. Bershadsky A. Geiger B. Safran S. A. (2002). Calculation of forces at focal adhesions from elastic substrate data: the effect of localized force and the need for regularization. *Biophys. J.* 83 1380–1394. 10.1016/S0006-3495(02)73909-X 12202364 PMC1302237

[B62] Seo H. S. Habas R. Chang C. Wang J. (2017). Bimodal regulation of Dishevelled function by Vangl2 during morphogenesis. *Hum. Mol. Genet.* 26 2053–2061. 10.1093/hmg/ddx095 28334810 PMC6075608

[B63] Stricker J. Aratyn-Schaus Y. Oakes P. W. Gardel M. L. (2011). Spatiotemporal constraints on the force-dependent growth of focal adhesions. *Biophys. J.* 100 2883–2893. 10.1016/j.bpj.2011.05.023 21689521 PMC3123981

[B64] Sucre J. M. S. Vickers K. C. Benjamin J. T. Plosa E. J. Jetter C. S. Cutrone A. (2020). Hyperoxia injury in the developing lung is mediated by mesenchymal expression of Wnt5A. *Am. J. Respir. Crit. Care Med.* 201 1249–1262. 10.1164/rccm.201908-1513oc 32023086 PMC7233334

[B65] Sun Z. Guo S. S. Fässler R. (2016). Integrin-mediated mechanotransduction. *J. Cell Biol.* 215 445–456. 10.1083/jcb.201609037 27872252 PMC5119943

[B66] Uehata M. Ishizaki T. Satoh H. (1997). Calcium sensitization of smooth muscle mediated by a Rho-associated protein kinase in hypertension. *Nature* 389 990–994. 10.1038/40187 9353125

[B67] Unbekandt M. Croft D. R. Crighton D. Mezna M. McArthur D. McConnell P. (2014). A novel small-molecule MRCK inhibitor blocks cancer cell invasion. *Cell Commun. Signal.* 12:54. 10.1186/s12964-014-0054-x 25288205 PMC4195943

[B68] van Soldt B. J. Qian J. Li J. Tang N. Lu J. Cardoso W. V. (2019). Yap and its subcellular localization have distinct compartment-specific roles in the developing lung. *Development* 146:dev175810. 10.1242/dev.175810 30944105 PMC6526715

[B69] Varner V. D. Gleghorn J. P. Miller E. Radisky D. C. Nelson C. M. (2015). Mechanically patterning the embryonic airway epithelium. *Proc. Natl. Acad. Sci. U.S.A.* 112 9230–9235. 10.1073/pnas.1504102112 26170292 PMC4522767

[B70] Vladar E. K. Königshoff M. (2020). Noncanonical Wnt planar cell polarity signaling in lung development and disease. *Biochem. Soc. Trans.* 48 231–243. 10.1042/BST20190597 32096543 PMC8969929

[B71] Vuga L. J. Ben-Yehudah A. Kovkarova-Naumovski E. Oriss T. Gibson K. F. Feghali-Bostwick C. (2009). WNT5A is a regulator of fibroblast proliferation and resistance to apoptosis. *Am. J. Respir. Cell Mol. Biol.* 41 583–589. 10.1165/rcmb.2008-0201OC 19251946 PMC2778165

[B72] Wada K. I. Itoga K. Okano T. Yonemura S. Sasaki H. (2011). Hippo pathway regulation by cell morphology and stress fibers. *Development* 138 3907–3914. 10.1242/dev.070987 21831922

[B73] Waters C. M. Roan E. Navajas D. (2012). Mechanobiology in lung epithelial cells: measurements, perturbations, and responses. *Compr. Physiol.* 2 1–29. 10.1002/cphy.c100090 23728969 PMC4457445

[B74] Weidinger G. Moon R. T. (2003). When Wnts antagonize Wnts. *J. Cell Biol.* 162 753–755. 10.1083/jcb.200307181 12952929 PMC2172824

[B75] Westfall T. A. Brimeyer R. Twedt J. Gladon J. Olberding A. Furutani-Seiki M. (2003). Wnt-5/pipetail functions in vertebrate axis formation as a negative regulator of Wnt/β-catenin activity. *J. Cell Biol.* 162 889–898. 10.1083/jcb.200303107 12952939 PMC2172822

[B76] Witze E. S. Litman E. S. Argast G. M. Moon R. T. Ahn N. G. (2008). Wnt5a control of cell polarity and directional movement by polarized redistribution of adhesion receptors. *Science* 320 365–370. 10.1126/science.1151250 18420933 PMC3229220

[B77] Wu X. van Dijk E. M. Ng-Blichfeldt J.-P. Bos I. S. Ciminieri C. Königsho M. (2019). Mesenchymal WNT-5A/5B signaling represses lung alveolar epithelial progenitors. *Cells* 25:1147. 10.3390/cells8101147 31557955 PMC6829372

[B78] Yang M. T. Fu J. Wang Y. K. Desai R. A. Chen C. S. (2011). Assaying stem cell mechanobiology on microfabricated elastomeric substrates with geometrically modulated rigidity. *Nat. Protoc.* 6 187–213. 10.1038/nprot.2010.189 21293460 PMC7183577

[B79] Yang W. Garrett L. Feng D. Elliott G. Liu X. Wang N. (2017). Wnt-induced Vangl2 phosphorylation is dose-dependently required for planar cell polarity in mammalian development. *Cell Res.* 27 1466–1484. 10.1038/cr.2017.127 29056748 PMC5717403

[B80] Yates L. L. Papakrivopoulou J. Long D. A. Goggolidou P. Connolly J. O. Woolf A. S. (2010a). The planar cell polarity gene Vangl2 is required for mammalian kidney-branching morphogenesis and glomerular maturation. *Hum. Mol. Genet.* 19 4663–4676. 10.1093/hmg/ddq397 20843830 PMC2972698

[B81] Yates L. L. Schnatwinkel C. Murdoch J. N. Bogani D. Formstone C. J. Townsend S. (2010b). The PCP genes Celsr1 and Vangl2 are required for normal lung branching morphogenesis. *Hum. Mol. Genet.* 19 2251–2267. 10.1093/hmg/ddq104 20223754 PMC2865378

[B82] Yates L. L. Schnatwinkel C. Hazelwood L. Chessum L. Paudyal A. Hilton H. (2013). Scribble is required for normal epithelial cell-cell contacts and lumen morphogenesis in the mammalian lung. *Dev. Biol.* 373 267–280. 10.1016/j.ydbio.2012.11.012 23195221 PMC3549499

[B83] Yin H. Copley C. O. Goodrich L. V. Deans M. R. (2012). Comparison of phenotypes between different Vangl2 mutants demonstrates dominant effects of the Looptail mutation during hair cell development. *PLoS One* 7:e31988. 10.1371/journal.pone.0031988 22363783 PMC3282788

[B84] Yu J. Chen L. Cui B. Widhopf G. F. Shen Z. Wu R. (2016). Wnt5a induces ROR1/ROR2 heterooligomerization to enhance leukemia chemotaxis and proliferation. *J. Clin. Investig.* 126 585–598. 10.1172/JCI83535 26690702 PMC4731190

[B85] Yuan K. Shamskhou E. Orcholski M. Nathan A. Reddy S. Honda H. (2019). Loss of endothelium-derived Wnt5a is associated with reduced pericyte recruitment and small vessel loss in pulmonary arterial hypertension. *Circulation* 139 1710–1724. 10.1161/CIRCULATIONAHA.118.037642 30586764 PMC6443444

[B86] Zhang K. Yao E. Lin C. Chou Y. Wong J. Li J. (2020). A mammalian Wnt5a – Ror2 – Vangl2 axis controls the cytoskeleton and confers cellular properties required for alveologenesis. *eLife* 9:e53688. 10.7554/eLife.53688 PMC7217702 32394892

[B87] Zhao B. Ye X. Yu J. Li L. Li W. Li S. (2008). TEAD mediates YAP-dependent gene induction and growth control. *Genes Dev.* 22 1962–1971. 10.1101/gad.1664408 18579750 PMC2492741

