# Supplementary material for: The Planar Polarity Component VANGL2 Is a Key Regulator of Mechanosignaling
Source: Front Cell Dev Biol. 2020 Oct 29;8:577201. doi: 10.3389/fcell.2020.577201 (PMC7658195; doi:10.3389/fcell.2020.577201)
Supplement: Supplementary file 1 [file Data_Sheet_1.PDF]

Supplementary Material

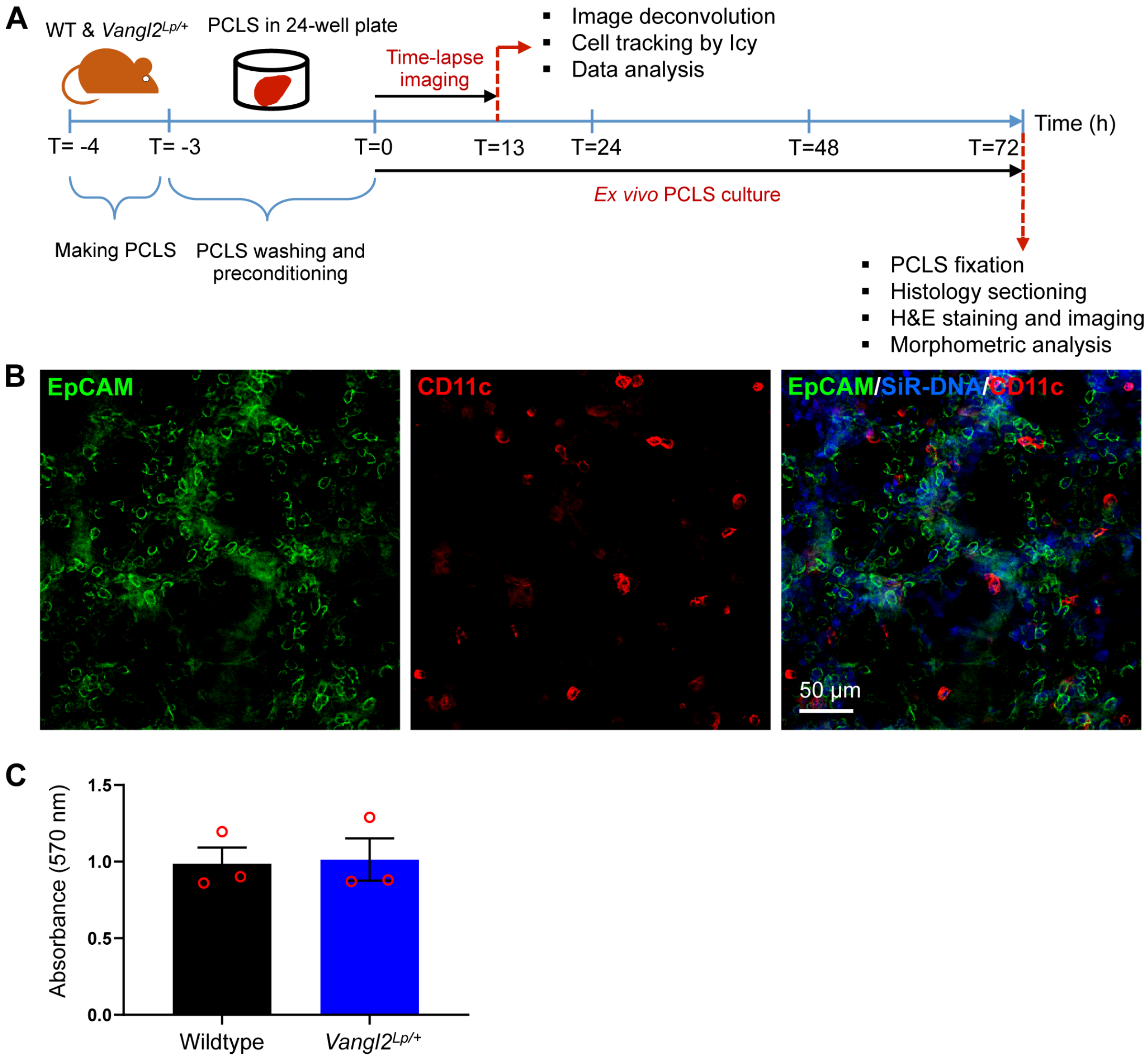

Supplementary Figure 1. Live imaging and *ex vivo* culture of wildtype and *Vangl2*$^{Lp/+}$ P3 mouse PCLS. (A) Timeline of PCLS slicing and experiments. **(B)** Deconvolved widefield, single plane, z-stack image of P3 PCLS stained with EpCAM-FITC (green, epithelial cells), CD11c-PE (red, macrophages) and SiR-DNA (blue, nuclei). n = 2 independent experiments. **(C)** MTT assay on P3 PCLS at 72 h of culture in serum-free DMEM. n = 3 independent experiments using three separate mice, quantification was from a single PCLS per group, per experiment; paired Student's t-test. Data are presented as mean ± SEM.

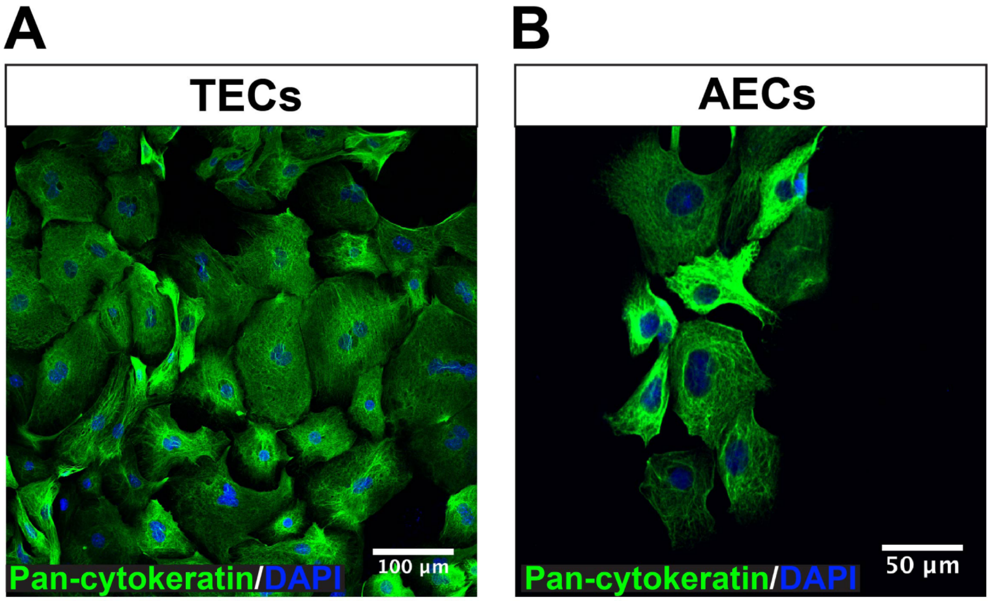

Supplementary Figure 2. Pan-cytokeratin staining in tracheal and alveolar epithelial cells. Cells were stained with epithelial cell marker, pan-cytokeratin (green). Nuclei were labeled with DAPI (blue).

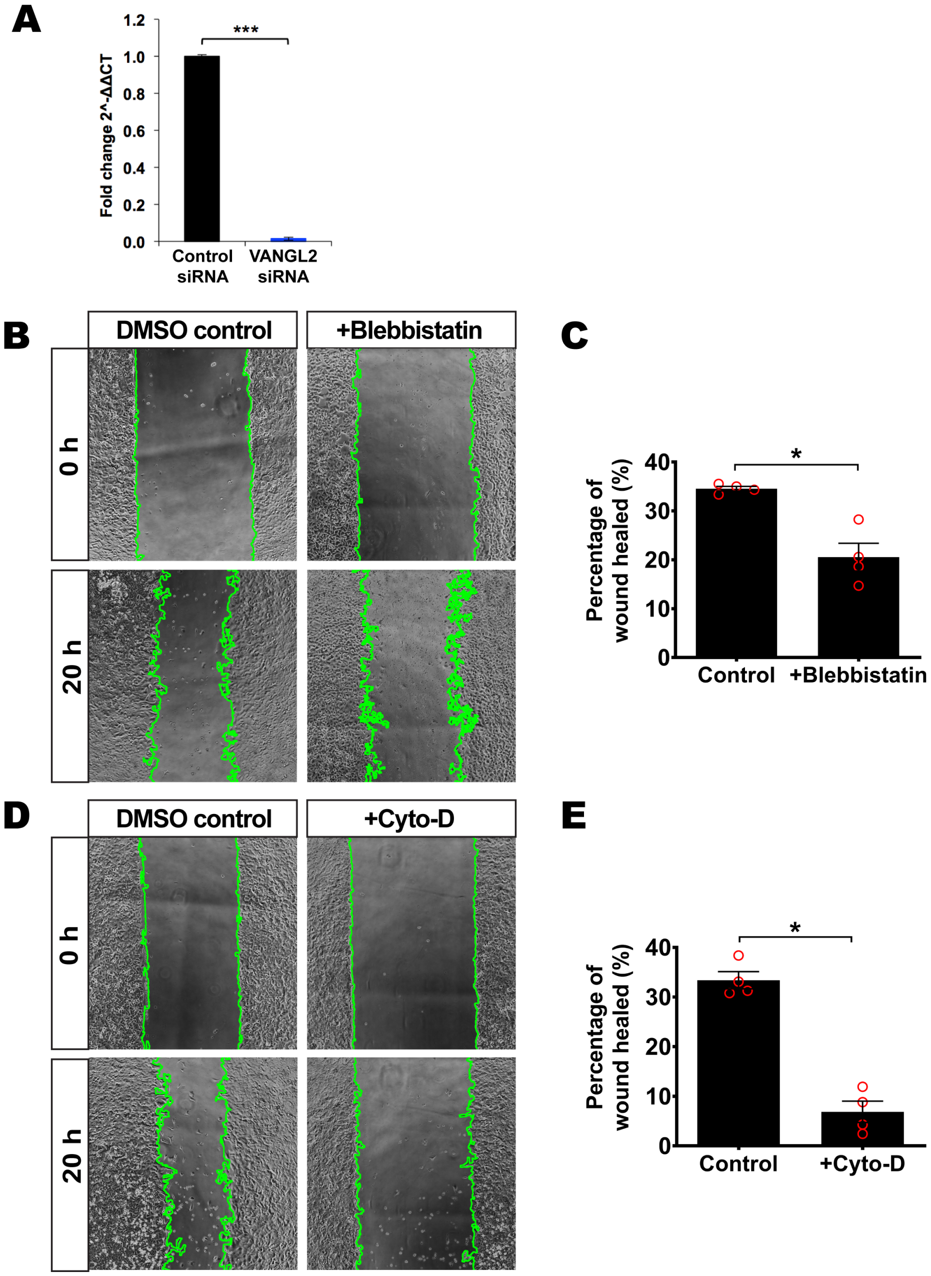

Supplementary Figure 3. Blebbistatin and cytochalasin D inhibit migration of A549 cells. (A) Quantification of *VANGL2* transcript levels by qRT-PCR showed a >90% reduction following *VANGL2*-siRNA treatment compared to control-siRNA treatment. n = 5 independent experiments; with triplicates for each experimental condition within one experiment. Data are presented as mean ± SEM; Mann-Whitney U test, ***p < 0.001. **(B)** Representative images showing DMSO control (left panels) and blebbistatin-treated A549 cells (right panels) at 0 h and 20 h post-scratch. Wound edges are indicated by green lines. **(C)** Percentage of wound healed measured at 20 h post-scratch in control and blebbistatin-treated A549 cells. **(D)** Representative images showing DMSO control (left panels) and cytochalasin D-treated A549 cells (right panels) at 0 h and 20 h post-scratch. Wound edges are indicated by green lines. **(E)** Percentage of wound healed measured at 20 h post-scratch in control and cytochalasin D-treated A549 cells. n = 4 independent experiments; three technical replicates for each treatment per experiment; each dot represents mean percentage of wound healed per experiment. Mann-Whitney U-test, *p = 0.029.

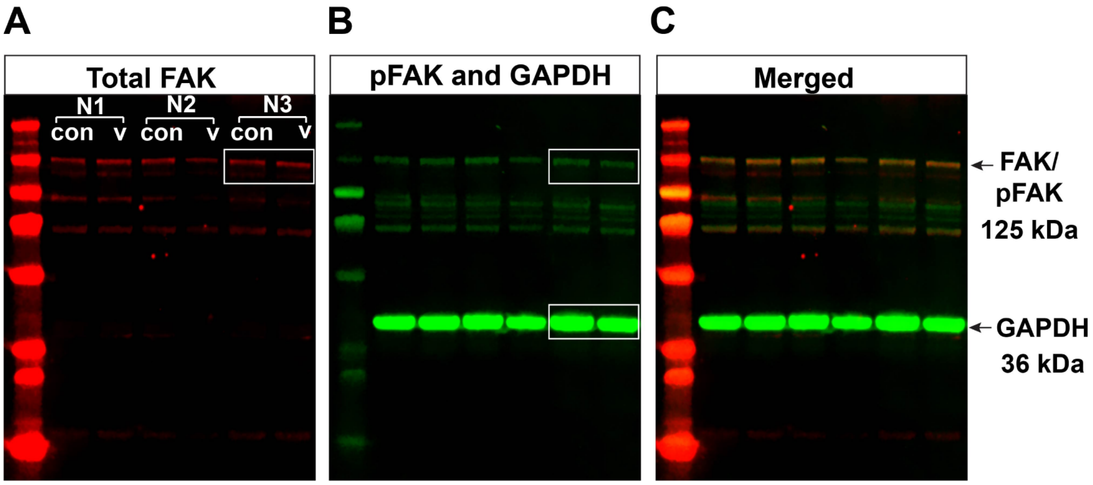

Supplementary Figure 4. FAK and pFAK in control- and *VANGL2*-siRNA transfected A549. (A-B) Whole western blots show protein lysates extracted from A549 cells transfected with control siRNA- (indicated by "con") or *VANGL2* siRNA (denoted by "v") from three independent transfection experiments (N1, N2 and N3), immunoblotted with (A) anti-FAK, and (B) anti-pFAK and anti-GAPDH (loading control). Total FAK was labeled with IRDye 680RD goat anti-mouse IgG secondary antibody; pFAK and GAPDH were labeled with IRDye 800CW goat anti-rabbit IgG secondary antibody. **(C)** Merged western blot shows the superimposed image of both 800 CW and 680RD channels. White boxes represent the lanes that were displayed in Figure 4P.

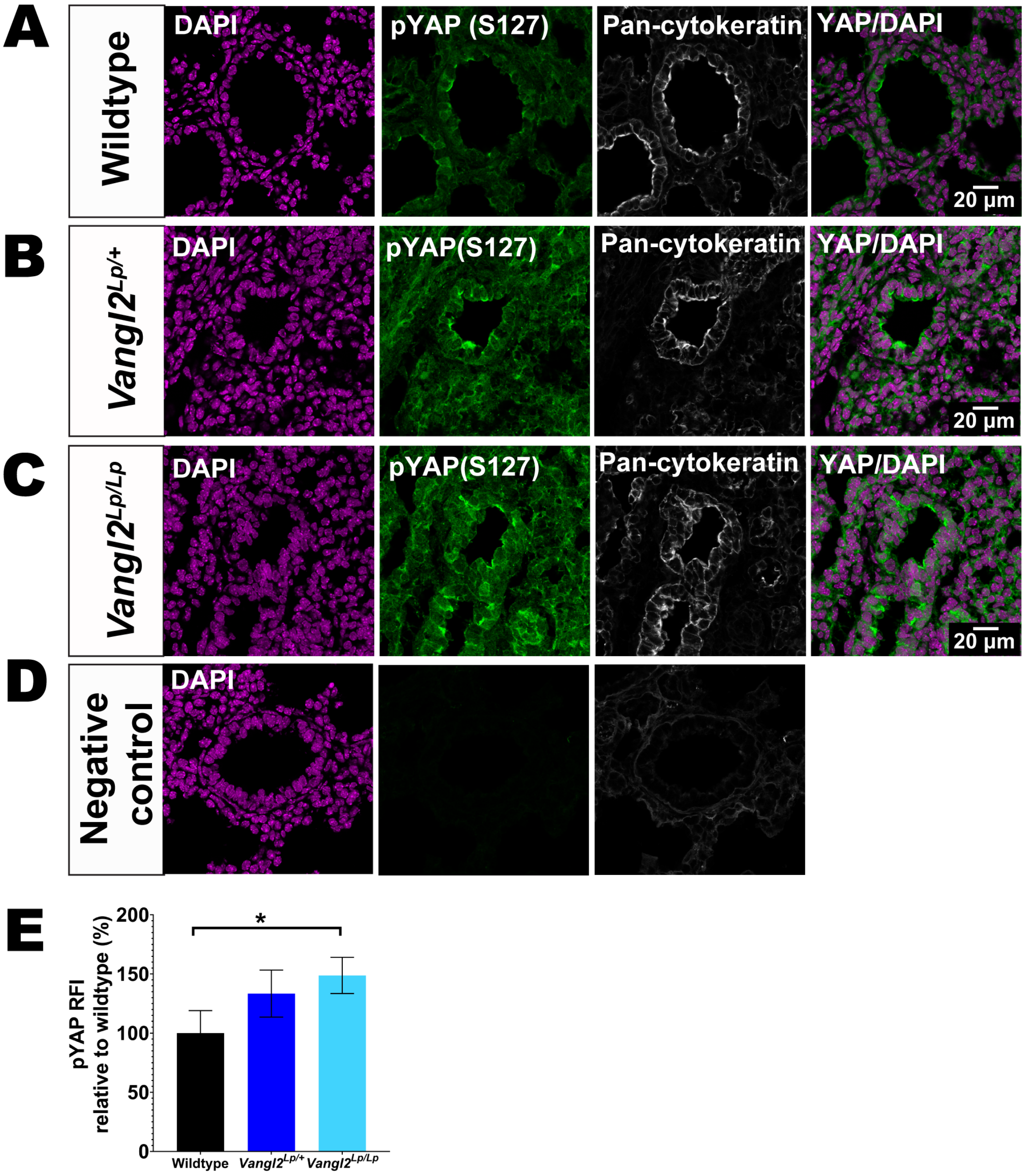

Supplementary Figure 5. Increased cytoplasmic pYAP in embryonic *Vangl2^{Lp}* airways. (A-C) Representative images show immunofluorescence staining for pYAP (Ser127) (green) in E18.5 wildtype, heterozygous *Vangl2$^{Lp/+}$* and homozygous *Vangl2$^{Lp/Lp}$* mouse lung cryosections. Sections were also stained with epithelial cell marker, pan-cytokeratin (grey) and nuclei were labeled with DAPI (magenta). **(D)** Negative controls with primary antibody omitted are shown. **(E)** Quantification of pYAP relative fluorescence intensity (RFI) in wildtype, *Vangl2$^{Lp/+}$* and *Vangl2$^{Lp/Lp}$* airways (presented as mean ± SEM). n = 3 technical replicates, 5 airways were analyzed per replicate. Kruskal-Wallis test, *$p < 0.05$.

Supplementary Video 1. Deconvolved 13 h time-lapse video of P3 wildtype PCLS. PCLS labeled with EpCAM-FITC.

Supplementary Video 2. Deconvolved 13 h time-lapse video of P3 *Vangl2*$^{Lp/+}$ PCLS. PCLS labeled with EpCAM-FITC.
